# Exploring past research to move forward: a scoping review of aims, outcomes, and recommendations in parental mental illness qualitative research

**DOI:** 10.3389/fpubh.2024.1427432

**Published:** 2024-10-16

**Authors:** Geneviève Piché, Gavin Davidson, Addy Dunkley-Smith, Anne Grant, Scott Yates, Darryl Maybery

**Affiliations:** ^1^Département de Psychologie et de Psychoéducation, Université du Québec en Outaouais, Saint-Jérôme, QC, Canada; ^2^School of Social Sciences, Education and Social Work, Queen's University Belfast, Belfast, United Kingdom; ^3^School of Rural Health, Monash University, Clayton, VIC, Australia; ^4^School of Nursing and Midwifery, Queen's University Belfast, Belfast, United Kingdom; ^5^School of Applied Social Sciences, De Montfort University, Leicester, United Kingdom

**Keywords:** parent, child, family, mental illness, outcomes, qualitative research, scoping review

## Abstract

**Introduction:**

As parental mental illness is a global public health concern, rigorous qualitative research is central to understanding families' experiences, needs and outcomes to inform optimal service provision in adult mental health and children's social services.

**Methods:**

The current review identified, appraised and synthesized international qualitative research exploring Families and Parent Mental Illness (FaPMI) research to determine the focus, findings and outcomes and to summarize the recommendations made about the direction of future research. Findings are classified according to outcomes for children, parents, and families.

**Results:**

While some children experienced positive outcomes from a parent's illness, most faced impacts on their social-emotional wellbeing, school performance, increased caregiving responsibilities, strained parent relationships, and lack of understanding about parental mental illness. Some family members endured abuse and struggled to adapt to an ill parent's unpredictable needs, with reluctance to discuss the situation. Parents found parenting challenging yet viewed having children as a protective factor. Future research should gather diverse perspectives, explore within-family factors and social environments, develop and test interventions, and address methodological issues like sampling.

**Discussion:**

This review highlights the centrality of qualitative data in comprehensively understanding and evaluating outcomes of parental mental illness on families and provides clear recommendations regarding future research.

## Introduction

The Prato Research Collaborative for change in parent and child mental health (1) recently indicated that research and interventions in this area have significant potential to improve outcomes and mental health of unwell parents and their families. The potential, positive impact of more effectively supporting families is considerable as ~23% of children have at least one parent with a history of mental health problems (2). It is also thought that approximately a third of individuals attending adult mental health services are parents (3) and a similar number of young people seeking child and adolescent mental health services have a parent dealing with mental illness (4) with 34% of such children exhibiting mental health symptoms in the high-risk range (5). Children that have a parent with a mental illness have a 2–13 times higher likelihood of developing mental health problems (6, 7, 81). Children in Families where a Parent has a Mental Illness (FaPMI) are also thought to be less likely to be school-ready (8), show higher rates of physical injury and are at greater risk of being taken into care (9).

Over the last 20 years there has been a 5-fold increase in family mental illness (FaPMI) research and it has recently been recommended that a set of quality outcome measures be developed to shape and assess practice in this field (10). The recommendation stemmed from a review of 50 quantitative studies of FaPMI parent, child and family focused research. The findings from that review showed considerable heterogeneity in terms of what is measured and instruments used, and a lack of focus on experiences of wellbeing [e.g., (11)] or caregiving (12, 13), impacts of intergenerational trauma (14), socioeconomic issues (15), and concerns regarding mental health literacy [e.g., (16)].

It is, however, unclear what kind of outcomes should be investigated or prioritized in future FaPMI research nor what areas have been investigated in past qualitative research. Qualitative literature offers a unique opportunity to delve into the experiences, needs, and challenges of families in a more profound and comprehensive manner than quantitative studies can achieve.

There have been a number of qualitative literature reviews in the FaPMI area. For example, a review by Cavanaugh et al. (17) focused upon mental health literacy websites for children of parents with a mental illness. Others have focused upon children's knowledge about what they know about their parents' illness (18), their experiences of living with a parent with a mental illness (19–21), or issues involved with access to help seeking for young people in such families (22). However, none of the reviews have focused upon what research has been undertaken previously nor what has been mentioned in publications as the focus for future research.

Exploring past qualitative research about the experiences of children, parents with a mental illness, and partners, including outcomes from intervention studies and recommendations for future research, will stimulate discussion about future directions. For these reasons, a scoping review was conducted to systematically map previous FaPMI research and determine the main outcomes that have been examined and the existing gaps in knowledge. This will inform recommendations for which outcomes should be investigated and measured in future research in this area.

### Research aims

The two aims of this scoping review were to: (1) scope and synthesize the research findings and outcomes in past qualitative research studies; (2) summarize any recommendations from past research about the direction of future research. This review of the literature scopes the findings and outcomes from qualitative FaPMI research since 2000. It is one component of a broader, international program of research seeking to determine and achieve a consensus on the most important research aims, outcomes and instruments to measure outcomes for families where a parent has a mental illness. Concurrently, a series of International Delphi studies with key stakeholders (e.g., young people, parents with lived experience, other family members, and mental health professionals) and a parallel review of the quantitative literature (10) are also being undertaken to determine through consensus the direction for future research in this field. The findings from the reviews will be combined with the first round Delphi results and then presented back to key stakeholders to obtain recommendations regarding a FaPMI outcome set. Altogether, the findings of the multi-study components will inform the aims of future FaPMI research including making recommendations about the methods and instruments (e.g., questionnaires) to be used to measure outcomes.

## Methods

A scoping review methodology [Joanna Briggs Institute JBI; (23)], modified with the Rapid Review Guidebook (24) was used to map and synthesize available literature targeting the review aims.

### Search strategy

The three academic databases chosen for this search were deemed the most relevant to this research area (Medline; International Bibliography of Social Sciences; PsycInfo). Gray literature searches were also conducted. A single librarian conducted all initial database searches, with no peer review of search strategies. Databases were searched on July 21, 2021 with follow-up searches conducted on May 15, 2023 and April 15, 2024 (by the same librarian) to identify any further relevant research in those periods.

The following terms were used: Outcomes and Parent^*^ mental illness OR illness OR disorder; COPMI or FAPMI; Famil^*^ mental health OR illness OR disorder; Famil^*^ psychiatry. Articles written in English, that contained the chosen terms either in the title, abstract, or keywords, were targeted. The search was limited to articles published from the year 2000, as research in this area has developed over that period. Manual searches of citations within reference lists of eligible studies were conducted by two of the researchers to identify additional studies potentially suitable for inclusion.

### Inclusion and exclusion criteria

Papers were included if they: (1) addressed experiences or outcomes of parent mental health for children and families; (2) studies were written in English in peer-reviewed journals; (4) studies were published in or after 2000. These results included qualitative, quantitative, review and data linkage papers—only the former were included here. Papers were excluded if they: (1) were quantitative, (2) were literature reviews, (3) addressed perinatal parent mental illness (as they involve different approaches to measurement, e.g., observation of parent-child interactions); (4) investigated parent-child prevalence estimates in mental health institutions; (5) were data linkage studies (e.g., outcome variables not established by the study authors); and (3) were editorial texts, commentaries, or opinion papers.

### Selection of sources of evidence

A two-step screening process was used: (1) screening of titles and abstracts; (2) screening of full-text articles. Duplicates were removed. Covidence, a web-based app for systematic reviews (25), was used by two of the researchers to independently screen each abstract. A third reviewer was consulted to settle any disagreement regarding inclusion or exclusion of a document. Next, full-text screening of the identified records was undertaken independently by two researchers, with disagreements settled by a third researcher (see PRISMA flow chart).

**Figure 1:**
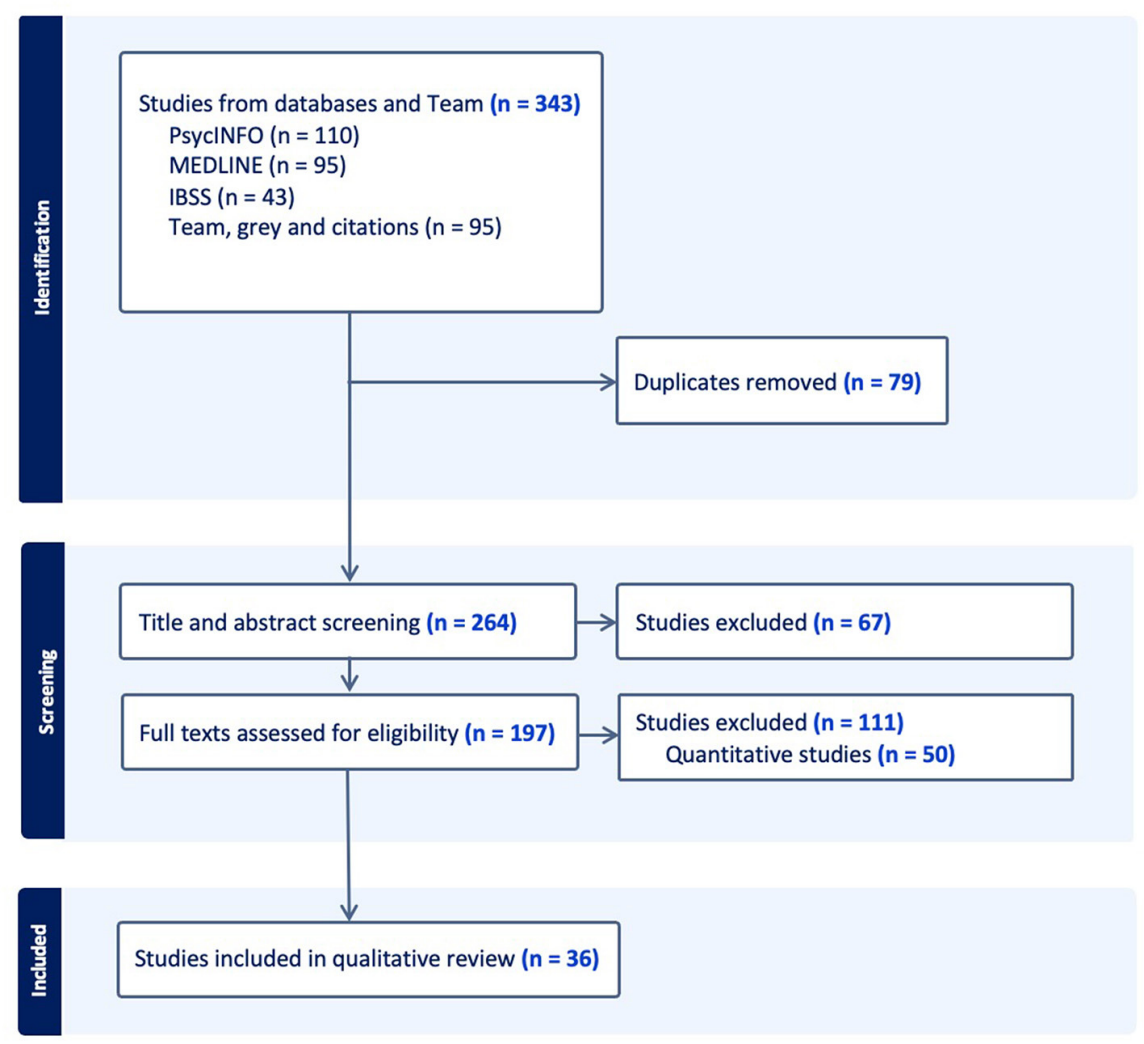
PRISMA diagram showing flowchart of data search and screening.

The PRISMA [Preferred Reporting Items for Systematic Reviews and Meta-Analyses] flowchart in Figure 1 generated by Covidence in May 2024 shows the process of screening and data extraction conducted in the review.

### Selection of records

The initial search identified 175 results (PsycInfo 94, Medline 50 and IBSS 31). The 2023 update identified a further 47 (PsycInfo 8, Medline 28 and IBSS 11), and the 2024 update another 26 (PsychInfo 8, Medline 17 and IBSS 1). An additional 95 potentially relevant articles were identified by the research team, giving a total of 343 results. Duplicates (*n* = 79) were removed, and 264 studies were then title-abstract screened. Figure 1 shows that the initial screening of 264 papers identified in the literature search resulted in 67 being evaluated as not relevant. The full texts of the 197 retained studies were then further screened for eligibility, resulting in 111 being excluded. Finally, 86 studies were assessed to be eligible for inclusion in the final literature review, of which 50 were quantitative and 36 were qualitative studies, the latter were included for review in this paper.

### Quality assessment of research

Two reviewers assessed the quality of the qualitative studies using the CASP Critical Appraisal Skills Program checklist, which includes 10 items around qualitative methodological indicators such as the study context, validity, quality of analytical methods, and rigor (see Supplementary Table 2) (82). The CASP is regularly used to assess the quality of qualitative papers (26). The quality of studies was assessed with Long et al. (27) revised version, by rating whether it met each criterion fully (“2 points”), partially (“1 point”), or not at all/can't tell (“0 point”). The total CASP score for all papers was used to categorize the methodological quality as either “high” (score = 20), “moderate” (16 ≤ score ≤ 19), or “low” (score ≤ 15) (28). Table 1 shows the quality assessment of the included studies in our scoping review.

**Table 1:** Description of the included studies.

**Study #**	**References**	**Country**	**Type of study**	**Sample**	**Participant group**	**Quality rating**
1.	Afzelius et al. (48)	Sweden	Interviews	Five PMI families, nine adults (five females), six children (four girls, two boys) aged 10–12 years. All had been involved in a family intervention.	Parents, partners, and COPMI	Moderate
2.	Bartsch et al. (49)	Australia	Focus groups	11 mothers (one father), 29–59 years old with present or past BPD.	Parents	Moderate
3.	Bartsch et al. (50)	Multi-country	Online survey open-ended questions.	106 clinicians—Australia (*n* = 65), the USA (*n* = 36), Canada (*n* = 2) and New Zealand (*n* = 2).	Clinicians	Low
4.	Bosch et al. (29)	The Netherlands	Interviews	18 (11 girls, seven boys) COPMI, aged 12–21 years.	Youth COPMI	Moderate
5.	Cudjoe et al. (30)	Ghana	Interviews	21 children aged 10–17 (13 girls, eight boys) who lived with a parent with MI.	COPMI	Moderate
6.	Cudjoe et al. (30)	Ghana	Interviews	13 children aged 10–17 (eight girls, five boys) who lived with a parent with MI.	COPMI	Moderate
7.	Davies et al. (22)	South Africa	Interviews	15 Health Care Professionals (HCP).	Clinicians	Moderate
8.	Drost and Schippers (31)	The Netherlands	Case study of weekly email interviews and textual analysis of participants chat sessions.	24-year-old female	Adult COPMI	Moderate
9.	Duncan and Browning (45)	New Zealand	Interviews	23 adult COPMI aged 24–61 years (four males, 19 females), 15 in relationships, five divorced/separated, three single/never married.	Adult COPMI	Low
10.	Fjone et al. (32)	Norway	Interviews	20 COPMI aged 8–22 years, one or both parents with mental health distress during the participant's childhood	COPMI	Low
11.	Foster et al. (54)	Australia	Interviews	14 COPMI aged 9–17 years (nine girls, five boys)	COPMI	Moderate
12.	Grove et al. (61)	Multi-country	Survey open-ended questions.	23 PMI researchers. Twelve females, seven males, four unknown. Australia (*n* = 6), Norway (*n* = 3), England (*n* = 2), United States (*n* = 2), the Netherlands (*n* = 2), Canada (*n* = 2), Italy (*n* = 1), Switzerland (*n* = 1), Finland (*n* = 1), and New Zealand (*n* = 1).	Researcher	Moderate
13.	Harries et al. (51)	United Kingdom	Interviews with	Eight parents with psychosis (five mothers and three fathers).	Parents	Moderate
14.	Hoadley et al. (52)	Australia	Survey open-ended questions.	22 parents aged 43–65 years, 83.3% two parent families), 12 COPMI aged 9–17 years (seven girls, five boys), six clinicians.	COPMI, parents clinicians	Low
15.	Isobel et al. (55)	Australia	Interviews and focus groups with	12 COPMI aged 9–17 years and three mothers, eight clinicians (six nurses and two OTs).	COPMI, parents clinicians	Moderate
16.	Knutsson-Medin et al. (60)	Sweden	Survey open-ended questions—mixed methods study	36 young people aged 6–17 years (17 girls, 19 boys).	COPMI	Low
17.	Marston et al. (53)	Australia	Telephone interviews	15 parents aged 30–49 years, 87.1% female after watching DVD.	Parents	Low
18.	Maybery et al. (58)	Australia	Survey open-ended questions	Children (*n* = 24) and parent (*n* = 18) dual diagnosis (parent) families not shown.	COPMI, parents	Low
19.	Mechling (33)	USA	Interviews	10 adult COPMI aged 18–25 years (seven females, three males).	Adult COPMI	Low
20.	Mordoch (34)	Canada	Interviews	22 COPMI aged 6–12 years (eight girls, 14 boys) who lived full or part time with a parent with a MI.	COPMI	Moderate
21.	Morningstar (35)	USA	Interviews	50 young adults aged 19–34 years, who were children of parents with mental illness	Adult COPMI	Low
22	Nevard et al. (59)	United Kingdom	Interviews	17 COPMI aged 6–17 years (11 girls, six boys).	COPMI	Moderate
23.	Nolte and Wren (46)	United Kingdom	Interviews	15 parents (13 mothers and two fathers) all had been diagnosed with severe and enduring mental health difficulties.	Parents	Low
24.	O'Brien et al. (57)	Australia	Interviews	Nine clinicians (three nurses, two psychiatrists/registrars, two social workers, and two occupational therapists) who had worked at acute units for at least the previous 12 months.	Clinicians	Low
25.	Power et al. (36)	Australia	Interviews	11 adults aged 18–51 years (eight female, three male) who were children of parents with mental illness	Adult COPMI	Low
26.	Radley et al. (37)	United Kingdom	Interviews	Five parents with a psychotic disorder (one father, four mothers), four children (one girl, three boys), three partners (two females, two males) and one grandmother.	Parents, partners, family members, COPMI	Moderate
27.	Reupert and Maybery (16)	Australia	Interviews	18 Expert COPMI program facilitators (Clinicians) from a Australian national public database of COPMI programs.	COPMI program facilitators	Low
28.	Tabak et al. (38)	Multi-country	Focus groups and interviews	Conducted with *n* = 96 clinicians (*n* = 50), parents with mental illness (*n* = 31), adult children and partners (*n* = 31) of parents with mental illness from England, Finland, Germany, Italy, Norway, Poland and Scotland.	Parents, adult COPMI, partners clinicians	Low
29.	Tanonaka and Endo (47)	Japan	Interviews	10 Adults aged 20–40 years (seven females, three males) who were children of parents with mental illness.	Adult COPMI	Moderate
30.	Trondsen (39)	Norway	600 posts—online COPMI self-help group	16 COPMI aged 15–18 years (15 females, one male)	Youth COPMI	High
31.	Trondsen and Tjora (44)	Norway	Interviews	13 COPMI aged 15–18 years (all females), participating in an online self-help group for adolescents with a parent with mental illness.	Youth COPMI	Moderate
32.	Van Parys et al. (40)	Belgium	Focus groups	18 adult COPMI aged 21–29 years (18 females, three males) who grew up with a depressed parent.	Adult COPMI	Moderate
33.	Van Parys and Rober (41)	Belgium	Interviews	Eight families (parents and partners) and 14 children aged 7–14 years.	Youth COPMI	Moderate
34.	Vetri et al. (56)	Canada	Survey open-ended questions	Eight parents (five mothers, three fathers), eight COPMI aged 7–11 years (four girls, four boys), and six psychosocial workers.	COPMI, parents, clinicians	Moderate
35.	Villatte et al. (42)	Canada	Photovoice group meeting	10 young adults aged 18–25 years old (nine females, one male) that have at least one parent with a mental illness.	COPMI	Moderate
36.	Widemalm and Hjärthag (43)	Sweden	301 comments from 35 forum threads on five Swedish COPMI Internet forums	Sample unknown, Mean age = 22 years, between 13 and 49 years.	Youth COPMI	Low

COPMI is the abbreviation for Children of Parents with a Mental Illness.

The quality of the included papers was either moderate (*n* = 20) or low (*n* = 15), with the exception of one high-quality paper. Detailed quality appraisal ratings are shown in Table 1. Significant limitations found in some studies were that they did not consider or discuss sufficiently the relationship between researcher and participants in their recruitment or analytical process, or that the data collection was not sufficiently rigorous (e.g., explicit description of the method, justification).

### Data charting and analysis

A data charting table was developed in Word by the researchers. The data was initially extracted by one researcher and the verbatim research aims were “cut and pasted” most commonly from the end of the introduction to the publications. The papers were then divided equally between two of the researchers and examined independently for future research recommendations. Suggestions for future research were then transferred verbatim into a table (see Supplementary Table 1). In addition to data directly related to our review questions, we charted data about country of origin, study design, sample characteristics (age, gender, sample size, and participant group).

Five of the papers were found to not have future research recommendations. These five were examined further by the alternate reviewer and three of those were found to have recommended future research in the text. Only two papers remained that did not indicate recommendations for future research. Recommendations for future research were commonly found in and generally at the end of the discussion, although at times in the abstract or introduction to the paper.

NVivo was used to facilitate the thematic analysis of the main findings and outcomes, from each study, which were then added to the data extraction table. Data were then synthesized through the writing of narrative summaries and interpretive analyses, guided by our review aim. Co-researchers were regularly consulted in the review process (during review design, data charting and analysis).

## Results

As shown in Table 1, 27 of the 36 studies included participants who had had a parent with a mental health problem when they were growing up. Ten of these included children or adolescents, nine included young adults or adult COPMI as study participants. Four studies focused only on parents, a further four that included families and/or parents and children, three studies with children, parents and clinicians and a further study that included parents, partners, adult COPMI and clinicians. Three studies included clinicians only and studies with one each of COPMI programme facilitator and researcher participants.

Nine of the studies were from Australia, four from the UK, three each from Norway, Sweden, Canada, and international collaborations, two from Ghana and the USA, two from the Netherlands and Belgium and one each from Japan, South Africa and New Zealand.

The qualitative studies included in this review pursued two main research aims, either around getting a better understanding of the experiences of parental mental illness or exploring the acceptability or effects of a preventive intervention.

### Findings and outcomes of parental mental illness research

Twenty-three of the 36 papers sought to explore the experience of parental mental illness from multi stakeholder perspectives in order to better understand the respective experiences of the parent's mental illness. Four main themes were identified: child and young people outcomes; family outcomes; parent outcomes; and intervention outcomes. Subthemes within each were identified and are illustrated below (see Table 2).

**Table 2:** Overview of the focus, findings, and outcomes of parental mental illness research.

**Children outcomes**	**Family outcomes**	**Parent outcomes**	**Intervention outcomes**
Socio-emotional outcomes	Unpredictability	Negative impacts of a mental illness on parenting	Communication
Increased responsibility/caregiving	Reluctance to talk	The relationship with their children as a positive and protective factor	Connecting with others
Insufficient understanding	Adjusting to the needs of the ill parent		Emotions and feelings
Impacts on the parent-child relationship	Physical or verbal abuse		Coping and personal skills
Social outcomes			Understanding the problem
School experiences			
Internal and external resources			

#### Child and young people outcomes

Six subthemes were identified from the analysis of the findings from 18 studies illustrating the experiences of parental mental illness from the child and young people's viewpoint: socio-emotional outcomes, increased responsibility/caregiving, insufficient understanding, impacts on the parent-child relationship, social outcomes, school experience, and resources.

Socio-emotional outcomes for children include having developed mental illnesses (e.g., anxiety disorders, eating disorders, depression), and most participants specifically talked about negative emotions and feelings (29–43, 60). Illustrations of this were shown by two studies (31, 60), where participants indicated the following socio-emotional outcomes:

“*I am terrified of my mother when she gets angry*” [(31), p. 59]

“*I worried about my mother. I worried about what condition she would be in when I came home from school*.” (Man, 28 years old) [(60), p. 749]

“*I felt indecisive, inadequate, and ashamed in front of my friends*. (Woman, 21 years old) [(60), p. 749]

“*I felt abandoned and lonely because no one explained what happened*”. (Woman, 20 years old) [(60), p. 749]

Nevertheless, several children and young adults reported that although their childhood was impacted by their parent's mental illness, they also felt a few positive socio-emotional outcomes. For example, participants stated they felt more mature (40, 42) and autonomous/independent (35, 42).

Nine studies underlined that parent mental illness frequently resulted in the child assuming greater responsibilities at home (35, 37, 38, 40–43, 60), for their families, “*managing everything*” (60), caring for their brothers and sisters, offering both practical (e.g., washing, cleaning, and cooking) and emotional support [e.g., hugging the parent, “saying the right supportive things at the right time” (41)] to their parents (60). Some participants report having “*lost their childhood*” [(35), p. 67]. One participant, Lucy, said: “*I always felt like I had to take care of my mom as a kid*” [(35), p. 68]. This “*reversal of roles*” was sometimes felt positively by children (pleased to be useful), but more generally felt like a heavy burden (39).

Participants—mostly adolescents—from seven studies reported insufficient understanding of mental illness in general and about their parent's specific mental illness. They underline that they were not well-informed, and that parent mental illness was not discussed in their family, leading them to many unanswered questions (34–37, 41, 43, 44).

“*I'd never experienced anything like this before, so I didn't really understand why I suddenly couldn't recognize my own mother*.” [(43), p. 1,604].

Five studies discussed the impacts that parent mental illness may have on the parent-child relationship (29, 35, 38, 42, 60). Some children reported that their relationship with their ill parent was generally too emotionally demanding (35), others underscored the superficial, forced relationship they had with their parents (29, 60); and many stated that they could never rely on their parent with a mental illness to support or comfort them during difficult times, or to motivate them to achieve in school (35, 38, 42).

“*I just went through a break-up, and I wish I could have called my mom and had her talk me through it. But I just can't. I mean, I could, but what would come out of her mouth would just stress me out even more. So yea, it's something I'm learning to deal with*” (Sophia) [(35), p. 57]

Six studies highlighted how parental mental illness impacted social outcomes, such as reduced time spent with friends and difficulties in romantic relationships (31, 35, 38, 39, 43, 45). For example, the teenage forum writers in Widemalm and Hjarthag (43)′s study commented that they had less time with their friends, often staying home because of fear of their parents harming themselves.

“*Several times when I was out, she called me crying and asked me to come home. I wanted nothing more than to be out with my friends and to forget about everything then. But the fear of her doing anything stupid to herself was too strong. At that time, I didn't have much leisure time. Always hurried home to make sure that mum was okay*.” [(43), p. 1,603]

In five studies, participants underlined the effects that their parent's mental illness had on their school experience (30, 37–39, 43). Participants explained that they felt generally uncomfortable in school, unable to focus on the classroom work (30, 39, 43), with “*continuous thoughts of how well their parents are doing back home*” [(30), p. 8], not wanting people to know about their parent's mental illness for fear of being stigmatized by their peers (30, 37). In two studies, participants underlined this even led them to stop going to school (38, 43).

Findings from eight studies illustrate the resources that children recognize as helpful to cope with their parent's mental illness (30, 32, 39–42, 46, 47). Child participants talked about the importance of internal resources, such as self-reflection, capacity to set limits toward the parent, and taking time for oneself (30, 32, 39–42, 46, 47). In this vein, some children reported the helpfulness of engaging in sports or physical activities [e.g., (41)], or developing artistic expressions (32).

“*I like to listen to music. I listen to the type of music where I can recognize myself… that describes things I feel and…. It is important for me to get time to listen to music. I listen to music through the night and walk around with music on, through the day. If I feel sorry, or I am frustrated or just bored, it's just to close the door, listen to really loud music, scream and shout and… just get it out*.” (Girl 15, 2006) [(32), p. 471].

Participants in eight studies also underlined the significance of external resources, such as their social support network from inside the family (siblings, extended family), the school (e.g., teachers), or the services (e.g., mental health professionals) (30, 32, 39–42, 46, 47).

“*What helps us feel better about the challenges of having a parent with a mental disorder is social support. It allows us to confide in each other during difficult times, to share our happiness and to dream together. My girlfriend and some of my friends offer me a lot of support in my daily life. (Victoria)”*. [(42), p. 8].

#### Family outcomes

The unpredictability of the family context, their family's reluctance to talk about mental illness, their adjusting to the needs of the ill parent, and experience of physical and/or verbal abuse from their parent were the four subthemes identified from the analysis of 11 studies reporting on the family outcomes. Five studies discussed the unpredictability of the family context when a parent has a mental illness (33, 34, 36, 38, 39), leading some children to have uncertain expectations concerning their parent (13, 34).

“*I felt like I was walking on egg shells around my mom or dad*” [(33), p. 213]

“*My mom has rapidly fluctuating depression. She has one completely normal day, and on the next she doesn't want to talk to anyone and just cries and rants*.” [(39), p.179]

Participants from five studies also reported on their family's reluctance to talk about mental illness, and the silence that surrounded their parental illness (33, 36, 38, 40, 41). Many families were hiding the parent's hospitalizations, suicidal attempts, and diagnosis (13), to avoid shame (38).

Both children and partners in two studies underlined the energy invested in adjusting to the needs of the ill parent, for example, to prevent conflicts (39, 48).

Finally, in two studies, some children from family environments where there is a parent with mental illness, named having experienced physical and/or verbal abuse from their parent (30, 37).

“*Sometimes she [parent with mental illness] insults me. She beats me but when she does, I just leave her to do it*” (Eric) [(30), p. 3,521]

“*When she [parent with mental illness] is here, she disturbs my sibling a lot. She is always shouting at them, they can't even concentrate on their homework*” (Christabel) [(30), p. 3,521]

#### Parent outcomes

Two main subthemes of negative impacts on parenting and general positive outcomes emanated from the analysis of the findings from nine studies illustrating the effects of parent mental illness on the parents themselves.

Five studies underlined the negative impacts of a mental illness on parenting. For example, parents with a mental illness and clinicians evoked impacts on the capacity of the parent to maintain discipline or discussed the general parenting skills deficits (49). Results of three studies also underlined the parent's difficulties in maintaining safe and stable environments, managing interpersonal boundaries, and having adequate empathic responsiveness (31, 49, 50).

“*I rely on the kids [aged seven and 3 years old] to try and calm me down. They are the parent and I'm the child. So when I get angry and I say ‘why haven't you told me…' and they say ‘mummy, I'm listening. Calm down' and then I'll calm down*”. [(49), p. 476]

The other important theme was the relationship with their children seen as a positive and protective factor, enhancing their capacity for adaptive parenting (30, 50), motivating them to behave better (49), and felt as a source of safety, comfort (51) and hope (37).

“*I was a mess before I had kids. I had [eldest son] and my life got… it completely changed my life*.” (Melissa) [(37), p. 352]

“*Observing my child grow through different stages and seeing her achievements. The pride of bringing such a wonderful child into the world and seeing how she has turned out*.” [(49), p. 476]

#### Intervention outcomes

In addition, 12 studies explored the acceptability or effects of a preventive intervention including the five subthemes: communication, connecting with others, emotions and feelings, coping and personal skills and understanding the problem.

Participants from five studies reported that the intervention helped them with communication, either by giving them the language necessary to communicate (16), or by helping them feel more comfortable sharing their experiences (44, 52, 53).

“*This was a very good way to start that conversation that I've never really known how to start. And I was so surprised how receptive the kids were and I keep underestimating kids*.” [(53), p. 142]

The results from six studies underlined that the intervention proposed helped the participants connect with others (16, 42–44, 54, 55). Participants from three studies evoked their feelings of belonging to a group of others just like them (42–44).

“*Also want to thank for a crazily good site. I was so happy (and touched) when I found it. I recognize myself in so many stories. And now, for the first time, I KNOW that I'm NOT alone*.” [(43), p. 1,605]

Participants from six studies underscored that the intervention/service offered, had positive impacts as it decreased their negative emotions. They felt normal (42, 44), supported (42, 43, 60), relief (16, 44, 53, 60), hope (42, 44), and less lonely (44).

“*I had wondered: Is this right? Is it normal? So I felt good to get confirmation that I am not alone in my situation, that many people are struggling with the same things, and all my reactions are normal. It was a relief that there wasn't anything wrong with me after all*.” [(44), p. 1,411].

Six studies reported on the effects of the intervention/services offered on the children and the family's personal and coping skills. Some participants from three studies underlined that they felt stronger and braver (54), capable of being proactive in their own lives (44) or contributing to other's wellbeing (43, 54). Also, results from two other studies report that the intervention helped them learn coping strategies (16, 56), such as “*Asking someone for help when you need it*.” [(56), p. 6]

Another main subtheme that emerged from the review, and more specifically underlined in five studies, was the helpfulness of the intervention/services in understanding either general mental illness, the parent's particular mental illness, or the impact that the mental illness could have on their family (16, 42, 52, 53, 57), and even in identifying issues that were “*difficult (P9), or not clear (P8) to the parents, or which were described as previously hidden (P8), in the background (P9) or which needed identification (P6)*” [(52), p. 49].

### Future research

The second aim of this review was to summarize the recommendations for future research. Examination of the 36 qualitative papers resulted in four themes (1) investigate multiple perspectives; (2) research in specific topic areas; (3) developing and testing interventions; (4) methodological issues and improvements to research designs (see Table 3).

**Table 3:** Overview of the main recommendations for future research.

**Multiple perspectives**	**Specific topics**	**Developing and testing interventions**	**Methodological issues**
Young people's voice: look at different age groups and profiles	Young people (including adult CoPMI)	Current and developing interventions	More prospective studies and trials
Voices of parents, carers/partners, or clinicians	Within family factors	Online interventions/support	Improvements to methods of enquiry
	Social environment		Problems and improvements in sampling
			Develop and use quality survey instruments to better measure

#### Future research to find multiple perspectives

Twelve studies proposed that future research should include multiple perspectives, and these proposals were analyzed into two subthemes of: “young people's voice: look at different age groups and profiles” and “Voices of parents, carers/partners, siblings, or clinicians.”

Eight studies emphasized the importance of including children, and children from different age groups and profiles to better capture the nuances and developmental differences in children's experiences and opinions (29, 34, 39, 44, 47, 51, 54, 56).

“*The involvement of children themselves in the evaluation of interventions ensures the evaluations are accessible, relevant and appreciated, which then ensures that the services made available to them are actually used (50).”* [(56), p. 9]

“…*Studies could include younger children and examine their guilt and shame feelings. They might not know a lot about the parental mental illness or do not understand it yet, and therefore their guilt and shame feelings might differ from youth who might have more knowledge about parental mental illness*.” [(29), p. 169]

“*I suggest that the gender issue should be explored in future research about participation in online and offline self-help groups, and when addressing the everyday experiences of children and adolescents with mentally ill parents*.” [(39), p. 185]

Eight papers discussed the importance of taking the perspective of different groups, including parents, partners of adults with a mental illness, fathers more specifically, siblings and clinicians. Three studies underscored the need to consider the experiences, challenges, and perspectives of parents, particularly partners of adults with mental illness, who often navigate complex caregiving roles (29, 30, 44). Three other papers emphasized that research into the impact of parenting on fathers with a mental illness is limited and that it's important that future research aim to better understand fathers' experiences, challenges, and contributions to family life with a parent with a mental illness (45, 49, 51). One paper highlighted the significance of including siblings in research on families with a parent with a mental illness. Siblings play a vital role in children's lives, and their perspectives can offer unique insights into family dynamics, relationships, and the impact of various factors on children's development and wellbeing (35). Finally, one study emphasized the importance for future research to consider the perspectives of clinicians who work directly with individuals and families affected by mental illness (50). Understanding clinicians' perspectives can help identify gaps in services, improve communication between professionals and families, and enhance the effectiveness of mental health interventions and treatments.

Overall, these papers collectively underscored the importance of inclusivity and considering a diverse range of voices and perspectives when exploring the experiences of families living with a parent with a mental illness.

#### Future research in specific topic areas

Twenty papers proposed that future research should investigate three specific subthemes: “young people (including adult COPMI);” “Within family factors;” and “Social environment.”

Across the 10 papers underlining the first subtheme “young people,” two papers emphasized the importance of considering personal factors such as attachment and past traumatic experiences, within the context of systematic assessments of children growing up with a parent with a mental illness (33, 45). A further two studies highlighted the significance for future research to aim at better understanding the unique experiences and needs of young people who have a parent with a mental illness, during their transition to adulthood (35, 42). Authors from three studies highlighted the need for more research around shame, guilt and stigma among families that have a parent with a mental illness (29, 32, 36). Finally, two papers discussed how future research should investigate the resilience process of children and families confronted by parental mental illness, and how interventions could help enhance resilience characteristics (29, 54).

Across the 11 papers exploring the second subtheme “within family factors,” six studies highlighted the need for future research to investigate issues around the quality, dynamics, and effects of the parent-child relationship within the context of parental mental illness (29, 39, 46, 50, 53, 58). Various dimensions of the parent-child relationship, including communication patterns, emotional bonding, attachment styles, and parenting practices, still need to be explored. Understanding the nuances of the parent-child relationship can inform interventions aimed at strengthening family bonds, promoting open communication, and supporting children's emotional needs during challenging times. Also, five papers underlined the importance of investigating the children's and families' experiences on parental mental health, but according to various types of mental illnesses (33, 49, 50, 58). Also, future research should look into the support needed according to the different illnesses (47).

Across the six papers exploring the third subtheme “social environment,” four studies discussed the need for future research on the role of social support and peer support, on children and families that have a parent with a mental illness (30, 42, 43, 54, 59).

“*Further research into the impact of social networking in peer support, the specific resilience characteristics enhanced by peer support, and the effects of peer support on other family members (e.g., parents and siblings) would add to the emerging evidence base on peer support programs*”. [(54), p. 66]

Another paper specifically recommended that future research be conducted on examining the role that culture plays in “*shaping how children understand and talk about their own families*” [(35), p. 182].

#### Developing and testing interventions

Nine papers proposed that future research should focus on *Developing and testing interventions*, and these proposals were analyzed into two subthemes of: “Current and developing interventions” and “Online interventions/support.”

First, authors from five studies recommended that more research be conducted to evaluate the acceptability, feasibility, and effectiveness, of current interventions, test and compare different educational modes, or propose new intervention strategies based on emerging research findings or clinical insights (16, 37, 50, 52, 57).

Also, four papers recommended that future studies investigate the effectiveness of online interventions and support for children or adolescents with parental mental illness (31, 43, 44, 54).

“*the body of research on online self-help groups has focused primarily on services for adult users and for adult caregivers of a child/partner suffering from illness*.” “…*more research is needed to determine the usefulness of online information and support for caregivers*.” [(44), p. 1,408].

#### Methodological issues and improvements to research designs

Finally, 15 papers discussed *Methodological issues and improvements to research designs*, around four main subthemes: “More prospective studies and trials;” “Improvements to methods of enquiry;” “Problems and improvements in sampling;” and “Develop and use quality survey instruments to better measure.”

Six papers underlined the need for more rigorous prospective studies and trials, investigating both short but also longer-term benefits or outcomes of interventions on children who have a parent with a mental illness (16, 48, 52, 54, 55, 60).

“*There is, however, a shortage of research showing the effect of these interventions in natural clinical contexts as well as of trials assessing parents' and children's outcomes, both in short- and long-term perspectives* (Schrank et al., 2015).” [(48), p. 70].

Four papers discussed the need to improve the methods of enquiry in future research (33, 41–43). For example, by using more qualitative or microanalysis methods (41), triangulation of perspectives (43), individual interviews or focus groups with children (41).

“*While analyzing family interviews, we learned to be careful not to overlook silences and other non-verbal cues. But many things are left unspoken and therefore the question remains: How to research concepts like ‘hiding worry' in an empirical way? Studying what is hidden or implicit is difficult because as a researcher you understand the words in a certain context, including complex non-verbal cues. Whereas, words are compelling in the context of research, the non-verbal issues are sometimes difficult to express in an article, let alone to objectify them*.” *(p. 342)* “…*our interpretation should be handled with caution and needs further exploration, for instance in other qualitative research projects*.” [(41), p. 342].

Six papers discussed about the need to improve our sampling methods (29, 35, 39, 52, 59, 61), to include more diverse samples, as well as larger, representative samples of participants.

“*Future researchers should also include larger samples of key stakeholders especially parents with mental illness, their children, and other family members*” [(61), p. 254].

Finally, three studies talked about the need for researchers to develop and use higher-quality survey instruments to better measure outcomes (29, 42, 61).

“*Researchers need to test outcomes of programs using scales with good psychometrics and control or comparison groups*.” [(61), p. 254].

## Discussion

This scoping review had two main objectives: (1) To scope and synthesize the research findings and outcomes in past qualitative research studies; (2) To summarize any suggestions from past research about the direction of future research.

Across the studies, the most salient outcome reported from child participants were socio-emotional outcomes, with many reporting mental health issues such as anxiety, eating disorders, and depression, along with negative emotions such as fear, guilt, loneliness, shame, and worry. These were also frequently reported as outcomes of participating in an intervention, as participants from six studies highlighted that the intervention or service they received had positive effects by helping them feel normal, supported, relieved, hopeful, and less lonely (42–44, 60). To our surprise, outcomes such as attachment and traumatic experiences were not frequently reported by participants in selected studies and were underlined by researchers as important areas for future research. Yet, in a recent parallel scoping review of quantitative studies in this area, results showed a similar trend, as none of the identified studies measured parent trauma, and only one group of researchers investigated trauma in young persons of parents with a mental illness (10). Attachment and trauma are multifaceted constructs that require specialized, often longitudinal, methods to assess accurately (62). This complexity may deter researchers from focusing on these outcomes. Yet, investigating attachment and trauma is crucial because both may have long-term implications for the mental health and development of children of parents with mental illness (63). Trauma, in particular, is a known predictor of future mental health issues, perpetuating intergenerational cycles of mental illness (64). By incorporating these outcomes into future research, we can better understand the holistic impact of parental mental illness on families and design interventions that support not just symptom management, but also long-term emotional wellbeing.

Second, other important reported outcomes of parental mental illness were on children's social and academic lives. Child participants reflected on how parental mental illness impacted their school performance, ranging from difficulties focusing in class to potential school dropout (30, 38, 39, 43). Participants also underlined having reduced time spent with friends and challenges in romantic relationships (31, 35, 38, 39, 43, 45). This theme of social connection emerges as a recurring outcome in qualitative studies exploring preventive interventions for children and families affected by parental mental illness. In three studies, participants described a sense of belonging to a community of peers facing similar challenges (42–44). At the same time, the role of external resources, such as social support or peer support, underlined in eight studies, was highlighted by some studies as a significant aspect that should be investigated more in future research (30, 42, 43, 54, 59). For example, future research should delve deeper into the mechanisms by which social support and peer connections contribute to positive outcomes for children and families affected by parental mental illness. Also, longitudinal studies are needed to explore whether social support networks persist after the intervention ends and how sustained connections might influence long-term outcomes. Moreover, with the rise of digital and online platforms for mental health support (65), there is potential for exploring how technology can facilitate social connections for families.

It is also not surprising that children's academic lives are impacted. Parental mental health problems have been linked to children's lower academic achievement in their final school year (66) and a parent's perinatal hospitalizations for mental health concerns has been associated with lower adolescent academic performance (67). This appears an important area for future research. Increasing attention is now being paid to children's mental health in schools in various countries (e.g., Northern Ireland, Canada) and guidelines are being developed for schools on how best to identify and support young carers. Further research could explore the impact of these recommendations.

Third, five studies underlined the negative impacts of a mental illness on parenting, specifically on the parent's difficulties with either: maintaining discipline (49), maintaining safe and stable environments, managing interpersonal boundaries, and having adequate empathic responsiveness (31, 49, 50). These impacts on the parenting skills of the parent with a mental illness echoes with the impacts reported by children, on their relationship with their parent. Children in five studies highlighted the emotional strain (35), artificiality (29), and lack of support (38) in their relationships with an ill parent (35). Some children even reported having experienced physical and/or verbal abuse from their parent (30, 37), others highlighted the impact on their caregiving responsibilities within the family (41, 60). One participant, Lucy, said: “*I always felt like I had to take care of my mom as a kid*” [(35), p. 68]. Hence, the parent-child relationship, particularly parenting skills, was a key outcome for both parents and children. At the same time, it is also noted by some studies, as a theme that should be investigated more in future research. Six studies stress the need for more research on the parent-child relationship during parental mental illness, including aspects like communication, bonding, attachment, and parenting (29, 39, 46, 50, 53, 58). Future research should explore how mental health symptoms interfere with a parent's capacity to be emotionally available and attuned to their child's emotions (68), as well as strategies for enhancing bonding in families affected by mental illness.

Fourth, children and partners of adults with partners of adults with mental illness emphasized the unpredictable nature of the family environment when one parent is affected, and the uncertain expectations regarding their parent (33, 34, 36, 38, 39), particularly considering that children generally underline being not well-informed about their parent's specific mental illness (37, 39, 41, 43). Participants in five studies disclosed that their families avoided talking about mental illness and kept quiet about their parents' struggles (13, 36, 38, 40, 41), with many families concealing hospitalizations, suicidal attempts, and diagnoses to evade shame (13, 38). Hence, children and families limited understanding of the parent's mental illness, and inherent unpredictability associated with this along with the various adverse socioemotional outcomes reported in many of the papers illustrate how important it is to focus and target mental health literacy, communication, and coping skills, as key outcomes of interventions; as also acknowledged in the studies reviewed. Notably, participants from five studies found the intervention beneficial for understanding mental illness, and aided them in communication, either by providing the necessary language or by making them feel more at ease in sharing their experiences (16, 44, 52, 53). One area that could benefit from further exploration is the role of stigma in perpetuating silence and avoidance around parental mental illness, as well as how interventions can address stigma to foster more open communication and emotional transparency within families.

Fifth, the qualitative studies identified in this scoping review reveal a nuanced understanding of the impact of parental mental illness on parenting and children. While negative impacts are acknowledged, there is also a recognition of positive outcomes for both parents and children, offering a refreshing perspective. Among the findings, parents from various studies reported that their relationship with their children served as a protective factor, motivating them in their general parenting (30), to behave better (49) and more generally it was for them a source of comfort and hope (37). Children, too, expressed positive experiences, noting that having a parent with a mental illness facilitated their personal growth toward maturity and independence (42). It's noteworthy to consider that effective family interventions capitalize on the strengths and positive aspects of individuals and their families. These findings not only shed light on potential targets for intervention, but also align with the principles of positive psychology interventions (69, 70), emphasizing the importance of leveraging inherent strengths to promote resilience and wellbeing within families facing mental health challenges.

More broadly, several researchers have emphasized the necessity for further research to thoroughly evaluate the acceptability, feasibility, and effectiveness of current interventions, including online interventions and supports for children with parental mental illness (16, 37, 50, 52, 57). Notably, there is a scarcity of studies assessing the acceptability or feasibility of interventions for COPMI or FAPMI, particularly those incorporating children's (56, 71) or young adult's (65, 72) perspectives, despite the recognized importance and benefits of such research (73–75).

Finally, studies highlight that more prospective studies and trials be conducted, as well as more high-quality research, including through improvements in sampling, and quality survey instruments. This finding supports previous suggestions of the need for high-quality study designs and methods (76) that have a focused group of psychological measures that are used by multiple authors (77) to ensure a greater homogeneity of measures to enable better interpretation of the problem and the effectiveness of clinical interventions (78). This issue and recommendation are further emphasized by the quality of papers in this study which showed that studies were either of moderate or low quality, except for one high-quality paper.

### Recommendations for future research and interventions

This review underscores the pivotal role of qualitative data in comprehensively understanding and evaluating outcomes of parental mental illness on families. By delving into the lived experiences and perspectives of individuals within these families, qualitative research provides invaluable insights that complement quantitative findings. However, the review also highlights the diversity in study designs across qualitative research in the field, which poses challenges for comparison and synthesis. There are clear opportunities for greater alignment of study designs, which would facilitate more effective comparison and synthesis across studies and settings, ultimately enhancing the robustness of research outcomes.

Furthermore, although some of the included studies focused on the outcomes of interventions, the majority centered around the general experiences of families rather than their specific experiences of support. This observation contrasts with quantitative studies and suggests future qualitative research could include targeted exploration of families' perspectives on effective support mechanisms (what works).

Moreover, while outcomes for children received considerable attention and depth of exploration, outcomes for parents or the entire family unit were comparatively less examined. This discrepancy underscores a gap in research focus that warrants attention in future studies to ensure a more holistic understanding of the impact of parental mental illness on family dynamics; particularly considering the increasing evidence of impact of parental mental illness on family functioning (79, 80).

The review also identifies several important factors for outcomes that were not extensively explored in the included studies. These factors encompass various domains such as economic difficulties and deprivation, experiences of discrimination or social exclusion, limited access or availability of effective support services, disrupted attachments, parent-child relationships, trauma experiences, child's illness or disability, and unemployment. These factors are reported in the wider literature as often associated with parental mental illness and may adversely impact all family members (81). Additionally, there's a call for more positively focused research, indicating a need to explore resilience, strengths, and coping mechanisms within families facing parental mental illness. Moving forward, recommendations for future research encompass the inclusion of multiple perspectives, such as those of children, fathers, and other family members, as well as efforts to promote the overall quality of research in the field through prospective studies and trials. Ultimately, involving families affected by parental mental illness in identifying research priorities emerges as a crucial step toward conducting meaningful and impactful research in this domain.

### Limitations

One of the limitations of this scoping review was the exclusion of papers specifically addressing perinatal mental illness and those focusing on the mental health workforce. Moreover, the search criteria were limited to research published in English, which introduces the possibility of language bias. Finally, it is important to acknowledge that qualitative data analysis, although a valuable tool for uncovering nuanced insights, is susceptible to interpretation and researcher bias. Despite efforts to maintain objectivity, the findings can be influenced by the perspectives of the researchers involved. As reported at the end of the paper, the authors are all from well-developed countries, predominantly with backgrounds in psychology or social work, working as academics and/or in a clinical role, with an interest in FaPMI research and practice.

## Conclusion

Research suggests that adverse outcomes for families affected by parental mental illness may be prevented or reduced by early identification of need and timely appropriate interventions. There has been a burgeoning amount of research in FaPMI and this study is important as it summarizes the scope of qualitative research from the last 24 years along with recommendations across those studies for future research. In combination with a recent review of the quantitative research over this period (10), the two reviews offer emerging recommendations and direction to move the field forward. The identification of child, parent, family and intervention outcomes along with future directions for research can also be used to inform policy, practice and service development.

## Author's note: reflexivity statement

Authors are all white women and men from well-developed countries (Australia, Canada, Northern Ireland, United Kingdom). All but one are parents and have had professional experience and training in psychology or social work, working as academics and/or in a clinical role, with an interest in Parent Mental health, Children of Parents with Mental Illness (COPMI) and FaPMI research and practice. Several of the team also brought a lived experience of parental mental illness perspective to the project.

## Data Availability

The original contributions presented in the study are included in the article/Supplementary material, further inquiries can be directed to the corresponding author.
