# Supplementary material for: Exploring past research to move forward: a scoping review of aims, outcomes, and recommendations in parental mental illness qualitative research
Source: Front Public Health. 2024 Oct 16;12:1427432. doi: 10.3389/fpubh.2024.1427432 (PMC11521813; doi:10.3389/fpubh.2024.1427432)
Supplement: Supplementary file 1 [file Table_1.pdf]

Supplementary Table 1: Verbatim research aims of 30 qualitative studies and their recommendations for future research.

Author, year and country	Study design, participants	Research aims	Recommendations for future research
Afzelius, M., Plantin, L., & Ostman, M. (2018). **Sweden**	Interviews with 5 PMI families, 9 adults (5 females), 6 children (4 girls) aged 10 to 12 years. All had been involved in a family intervention. **Families including COPMI Parent**	*The aim of this study was to explore how families in which a parent is diagnosed with a mental illness and supported by family interventions in a natural setting in the psychiatric services experience their situation. An additional aim was to examine the support provided by family interventions and how family members experience this support* (p.72).	*"There is, however, a shortage of research showing the effect of these interventions in natural clinical contexts as well as of trials assessing parents' and children's outcomes, both in short- and long-term perspectives (Schrank, Moran, Borghi, & Priebe, 2015)." (p. 70).*
Bartsch, D. R., Roberts, R. M., Davies, M., & Proeve, M. (2016). **Australia**	Focus groups with 11 mothers (1 father), 29-59 years old with present or past BPD. **Parents**	*The aim of the present study was to…exploring the lived experiences of parents with a diagnosis of BPD. Furthermore, there are no known investigations into these parents perceptions about the accessibility of parenting supports within the Australian context.* (p.473).	*"Further research should be attempted with parents with a diagnosis of BPD in different community and service settings. Furthermore, despite the researcher's efforts, the study was only able to recruit one father to participate in the interviews. Research into the impact of parenting on fathers with a diagnosis of BPD is limited and warrants more attention". (p. 478).*
Bartsch, D. R., Roberts, R. M., Davies, M., & Proeve, M. (2015). **International (see design)**	Online survey of open-ended questions. 106 clinicians from Australia (n = 65), the USA (n = 36), Canada (n = 2) and New Zealand (n = 2). **Clinicians**	*This study aimed to gather clinical opinions from a diverse range of mental health clinicians who had experience working with families where a parent had a diagnosis of BPD. First, opinions were sought on potential problems faced by parents with a diagnosis of BPD. Second, views on the effectiveness of available parenting resources, supports and interventions for parents with a diagnosis of BPD and their children were also explored.* (p.115).	*"More specifically, these authorsproposed that inconsistencies in emotional socialisation, as well as discipline and monitoring, could result in an invalidating environment, which may increase the risk that a child will develop their own emotional regulation difficulties. Research is still needed to evaluate and confirm this hypothesis.(p. 123). Zalewski et al. (2015) recently explored the feasibility of modifying DBT to incorporate parenting skills by interviewing mothers with a diagnosis of BPD who were currently in treatment. Mothers noted a number of potential benefits to this approach including commonality with other mothers, children learning skills and increased confidence as a parent. However, they also reported a number of challenges such as fear of being judged and fear of exposing their children to other parents with a*

Author, year and country	Study design, participants	Research aims	Recommendations for future research
			diagnosis of BPD. Whilst Zalewski et al. (2015) demonstrated that mothers with a diagnosis of BPD were open to having parenting skills integrated into their current psychiatric treatment, there is currently limited research demonstrating the efficacy of this approach. (p. 123). Furthermore, the development of future treatment programmes could also be informed by factors associated with adaptive parenting amongst parents with a diagnosis of BPD. However, future research on this topic is required. (p.124) It is recommended that this research be extended to include in-depth interviews with clinical staff.(p.124). Nevertheless, future research should also interview parents with a diagnosis of BPD about their perspectives relating to parenting challenges to see if they are consistent with clinicians' views and whether new themes emerge.(p.124). Whilst the present study touched on service provision there has yet to be research into clinicians' attitudes towards working with families where a parent has a diagnosis of BPD".(p.124).
Bosch, A., Riebschleger, J., & van Loon, L. (2017). **Netherlands**	Interviews with 18 (7 boys, 11 girls) COPMI, aged 12-21 years. **Youth COPMI.**	*Therefore, the present study aims to enhance knowledge about guilt and shame of COPMI youth, and the extent they report that such feelings may influence their relationship with parents.* (p. 160).	*"Future research should take this knowledge into account when examining feelings of guilt and shame in COPMI.*<br>o *For example, studies could include younger children and examine their guilt and shame feelings. They might not know a lot about the parental mental illness or do not understand it yet, and therefore their guilt and shame feelings might differ from youth who might have more knowledge about parental mental illness.*<br>o *... future studies could expand the conceptual base to include examining youth reports of positive feelings toward parents and resilient behavioral adjustments." (p. 169).*<br>*"Other informants, such as parents, may also inform about the parent-child relationships. Future research also could include the perspective of the parent. Finally, the sample was not demographically representative for the general Dutch population.*

Author, year and country	Study design, participants	Research aims	Recommendations for future research
			Future research should include a wider ranging sample of socioeconomic status."(p. 169).<br>*"Finally, the findings can be used to inform future survey studies. For example, the findings can provide input to develop specific questionnaires to better quantitively measure feelings of guilt and shame in COPMI". (p.170)*
Cudjoe, E., Tam, C., Effah, D., Amegashie, E., & Tweneboah, A. (2022).	Interviews with 21 children (aged 10-17) who lived with a parent with MI. **COPMI**	This study aimed to explore the phenomenon of what it is like in the family for children to live with a parent with mental illness. (p.3517)	*"The analysis considered here involved a somewhat smaller categorisation of the family including the parent with mental illness, parent without mental illness and siblings. Meanwhile, the family setting may extend to grandparents, uncles, aunts and several classifications of a kinship network. It is particularly so for children whose parents have mental illness where extended family members are integral to providing support. Thus, further studies may include a broader family network. This can also broaden the target for family-focused interventions to enable healthcare professionals to have a strong system of support to utilise in their practice". (p. 3525)*
Cudjoe, E., Tam, C., & Chiu, M. (2023).	Interviews with 13 children (aged 10-17) who lived with a parent with MI. **COPMI**	This study explored what children living with a parent with mental illness think about their interactions at school. (p.2)	No recommendations for future research
Dean, L., Buechner, H., Moffett, B., Maritze, M., Dalton, L., Hanna, J., Rapa, E., Stein, A., Tollman, S., & Kahn, K. (2023).	Interviews with 15 Health Care Professionals (HCP). **Clinicians**	This study aims to: (1) explore the experiences and perceptions of HCPs about beliefs and attitudes towards mental illness in their communities, (2) explore the experiences and perceptions of HCPs around families talking with children about a parent's mental illness, (3) investigate HCPs' perceptions of the obstacles and facilitators to communication with children about parental mental illness and where and	*"Further work is required to understand the beliefs and attitudes about communication with children regarding mental illness amongst community members (e.g. religious leaders, educators, general public) who do not have a professional role in healthcare. The perspectives and role of community leaders (including faith and traditional healers) regarding communication with children must be explored to inform an appropriate strategy to facilitate communication with children. This must also include the opinions of parents and children with experience of parental mental illness about what is needed to support their families".* (p. 9).

Author, year and country	Study design, participants	Research aims	Recommendations for future research
		with who these conversations should be initiated. (p.3)	
Duncan G & Browning J (2009). **New Zealand**	Interviews with 23 adult COPMI. 4 men and 19 women age ranging from 24 to 61. Thirteen were married, two in common-law marital relationships, five divorced or separated and three single or never married.<br>**Adult COPMI**	*…understandings of the complexity and variety of experiences, and the shared themes, running through the lives of the people concerned, from their own points of view. The present study is qualitative and interview-based. It elicits narrative insights from participants that illustrate outcomes in adult life for children of parents with schizophrenia*. (p.79).	*"Most current research concerns mothers with mental illness and their children. More research could focus on the father with mental illness and his children. .(p.84).*<br>*The present research method did not include an assessment of the adult attachment styles of the participants, and so no estimate can be made of the frequency of insecure adult attachment in this group, let alone any specific observations about ambivalent or avoidant styles. It does suggest, however, that future research could fruit fully include such systematic assessments, alongside qualitative narrative findings". (p. 84).*
Drost, L. M., & Schippers, G. M. (2015). **Netherlands**	Structured weekly email interviews and textual analysis of participants chat sessions. Case study 24 year old female COPMI<br><br>**Adult COPMI**<br>.	*The aim of the present study was to depict one user's views on her situation and on the online preventive support that was offered to her. It was thought that doing so would (a) expand our understanding of the key variables that influence COPMI's attempts to seek support and (b) determine whether a targeted website could help to improve COPMI's ability to cope with their circumstances and find professional help.* (p. 54-5).	*"In future, additional analyses should be undertaken to determine how online communication between young people and prevention professionals might influence consultation processes and alter healthcare outcomes (Harvey et al., 2008)". (p. 63)*
Fjone, H. H., Ytterhus, B., & Almvik, A. (2009). **Norway**	Individual interviews with 20 young people aged 8-22 years - each with one or both parents who suffered from mental	*The intention of this article is to increase our knowledge about what it is like to grow up with parents with mental health distress by examining how children who live with parents suffering from this health issue present them*	*"Children's apprehension of parents blaming themselves in these parental meetings or at school events creates feelings of fear and shame that the children try to avoid by not bringing the parent into the school setting…The social context in parental meetings requires the participation of mothers and/or fathers. Children do feel different from the others if the parent does not show up. It*

Author, year and country	Study design, participants	Research aims	Recommendations for future research
	health distress during the interviewee's childhood. **Youth COPMI**	*selves so as to avoid stigma and self-stigmatization and to be viewed as 'normal'.* (p.462).	*becomes a situation of 'damned if you do, and damned if you don't'. This might become a topic for further investigation » (p. 473).*
Foster, K., Lewis, P., & Mccloughen, A. (2014). **Australia**	Interviews with 14 children/adolescents 9–17 years of age, 5 boys and 9 girls. **Youth COPMI**	*Given the limited knowledge on children/adolescents' subjective experiences of peer support where parents and/or siblings have mental illness, this study aimed to provide valuable insights into children/adolescents' perspectives on the ON FIRE program.* (p.62).	*"Given the range of developmental stages of young people in the program, consideration could be given to implementing age-specific program activities tailored to particular groups (e.g., 8–12 and 13–17 years). Consideration could also be given to construction of a virtual meeting place for 13- to 17-year-olds—a website facilitated by program staff which includes regular blogs or chat rooms to discuss issues relating to mental health and illness and to connect outside the program. (p.65).*<br>*There is a lack of evidence on the ongoing effects of peer support. Longitudinal research that follows up peer support participants, and investigates the long-term impact of peer support in young people's lives, is recommended. Further research into the impact of social networking in peer support, the specific resilience characteristics enhanced by peer support, and the effects of peer support on other family members (e.g., parents and siblings) would add to the emerging evidence base on peer support programs". (p. 66).*
Fudge, E., & Mason, P. (2004). **Australia**	2 focus groups and 9 individual interviews with 33 children (aged 7-12) and 25 young people (aged 13-20). **Youth COPMI**	*This paper describes one of the consultation phases where young people in the 7 to 20 year age range were asked to comment, via focus groups and peer interviews, on issues raised in an early version of the document.* (p.1).	No recommendations for future research
Grove, C., Riebschleger, J., Bosch, A., Cavanaugh,	Qualitative survey of 23 PMI researchers. 12 females, 7 males, 4 unknown.	*Despite a growing literature focus on the needs of COPMI and emerging COPMI program data, there remains a gap in the research knowledge base*	*"Future researchers should also include larger samples of key stakeholders, especially parents with mental illness, their children, and other family members. There is a need to attention to differences and similarities within and among people from*

Author, year and country	Study design, participants	Research aims	Recommendations for future research
D., & van der Ende, P. C. (2017). **Internationa l (see countries in design).**	Australia (n = 6), Norway (n = 3), England (n = 2), United States (n = 2), the Netherlands (n = 2), Canada (n = 2), Italy (n = 1), Switzerland (n = 1), Finland (n = 1), and New Zealand (n = 1).	*about the specific kind of information that COPMI may need. …ask international experts on parental mental illness to participate in a survey to express their recommendations of the mental health literacy knowledge needs of children of parents with a mental illness*. (p.251).	*different countries of the world. Researchers need to test outcomes of programs using scales with good psychometrics and control or comparison groups".(p.254).*
Harries, C. Smith, D., Gregg, L., Allott, R., & Wittkowski, A. (2023).	Interviews with 8 parents with psychosis (5 mothers and 3 fathers). **Parents**	Thus, we sought to explore the experiences of parents who experience psychosis. The primary aim was to explore how these parents experienced parenting, the meanings they assigned to their experiences, and the support offered to them. We specifically addressed the question 'What is it like to be a parent who experiences psychosis?'. (p.3)	*"Future research should aim to explore the experiences of broader samples of fathers who experience psychosis.*<br>*Future studies should explore the experiences of parents with children in discrete age brackets.*<br>*Future research should replicate the findings across mothers and fathers living in different socio-political contexts and health care systems internationally before the study findings can be considered transferable to other contexts..." (p. 14)*
Hoadley, B., Falkov, A., & Agalawatta, N. (2019). **Australia**	Self-report questionnaire with 15 parents (Mean=51.5, 43–65 years, 83.3% 2 parent families), 8 young people (Mean=13.1, 9–17 years, 58.3% female), 6 clinicians. **Family members and clinicians**	*This pilot study will describe service user and clinician feedback concerning a single-session family focused intervention (TFM) in a public-sector Child and Youth Mental Health Service (CYMHS). This study focuses on service user and clinician reports about the helpfulness/unhelpfulness of the intervention and also seeks to generate data through open ended scoping questions.* (p.45-46).	*"Preliminary data indicate the acceptability of this single session intervention using The Family Model in a small sample of service users and clinicians in a public sector mental health setting. Further work is required to demonstrate feasibility and clinical utility in larger samples and different settings. (p.44 abstract). Further research would help to clarify the key ingredients and the mechanisms for achieving (and sustaining) positive change (p.52)(DM added - referring to a single session intervention using the family model). An area for future evaluation will be to better delineate indications and scope for use of TFM as a standalone approach, as an adjunct to existing treatment and its use as a model for brief (repeated session) intervention. (p.52). Clinical*

Author, year and country	Study design, participants	Research aims	Recommendations for future research
			outcomes for service users (with complex as well as less severe difficulties) and parents/families need to be evaluated in the short and long term…..Further training and upscaling to include all multidisciplinary team members, and the development of accessible learning materials to assist with workforce capacity building, would support future research opportunities". (p.52).
Isobel, S., Pretty, D., & Meehan, F. (2017). **Australia**	Interviews and focus groups with 12 young people (Mean=13, 9-17 years) and 3 mothers, 8 clinicians (six nurses and two OTs). **COPMI** **Parents Clinicians**	*To explore the meaning and experience of participation in a biannual child focused program delivered by AMH services to the children of clients of the service; from the perspectives of children, parents and mental health clinicians.* (p. 134).	*"Other studies on family-focused practice have identified that the gap between knowledge and practice in family centred practice (Maybery et al., 2016), which may be overcome by organisational support and a family orientated philosophy (Grant & Reupert, 2016). This school holiday program is an important example of an AMH service demonstrating to staff, families and children th eir commitment to family-focused practice. The effects of this on families and the wider workforce are worth further consideration. (p.143). Further longitudinal research is required to identify the specific benefits of his school holiday program for different stakeholders". (p.144).*
Knutsson-Medin L, Edlund B & Ramklint M (2007). **Sweden**	Written comments to questionnaire in mixed methods study, 36 young people (Mean=16.4 years, 17 female and 19 male). **Youth COPMI**	*The aim of this study was to follow up children of psychiatric inpatients and examine their experiences of growing up with a mentally ill parent and their opinions concerning their previous contact with psychiatric services, the extent of this contact, and their opinions about the deficiencies and merits of the services.* (p.745).	*"[In spite of good social adjustment, participants in this study reported increased levels of mental health service utilization. If they had received adequate help in coping with the burden of parental disease, they might have experienced fewer psychiatric symptoms themselves.] Studies of interventions are needed, particularly prospective studies that follow up these children from childhood to adult life". (p.751).*
Marston, N., Maybery, D. & Reupert, A. (2014). **Australia**	Telephone interviews with 15 parents between 30 and 49 years, 87.1% female after watching DVD. **Parents**	*The following presents an evaluation of the 'Family Focus' DVD with a group of volunteer parents. This research sought to answer the questions; how do parents use the 'Family Focus' DVD, if at all, do they include other family members in its utilization, and does this family led*	*"In addition, future evaluation needs to identify long term outcomes, with particular reference to sustained communication changes and the impact of these upon family dynamics, help seeking behaviour, parenting confidence and other basic tasks of family functioning. Children's outcomes in the long term also need to be measured" (p.144).*

Author, year and country	Study design, participants	Research aims	Recommendations for future research
		approach lead to any attitudinal and/or behavioural changes following exposure? (p. 137).	
Maybery, D., Reupert, A., & Goodyear, M. (2015). **Australia**	Children (n = 24) and Parent (n = 18) responses to open question regarding goal setting. Dual diagnosis (parent) families not shown. **COPMI Parents**	*This research examines the goals set by families with parents with MI and those families where a parent has a DD (a diagnosis of mental health and a substance abuse disorder). Specifically, the study sought to determine:* • *The most prominent goals set and achieved by family members where a parent has a MI.* • *The most prominent goals set and achieved by family members where a parent has a DD.* • *The similarities and differences in terms of goals set, and goals achieved, for parents and children from the different families.* (p. 356).	*"Another significant finding is that while family connectedness (including a focus on enhancing family interactions and family problem solving) was an important goal established for parents and children across both family types, relative to other goals, achievement was low (with the exception of children whose parents had a DD). While there have been repeated calls to support the relationships between family members where a parent has a mental health or substance abuse problem (e.g. Nicholson 2010), this might well be a difficult and long-term goal to achieve. Why children whose parents have a DD progressed reasonably well in this goal is unclear and requires follow up investigation". (p. 362).*
Mechling, B. (2016). **USA**	10 adult COPMI (18-25 years), 7 females and 3 males. **Adult COPMI**	*One, open-ended question was asked to elicit a description of the participant's experience of growing up with a parent suffering from depression: "Describe for me what your experience was like while growing up with your depressed parent?" And then a later cue was used: "And how has this experience affected you today?"* (p.213).	*"Few studies regarding growing up with a depressed parent in the home have been done within the science of nursing. Few studies regarding children of mentally-ill parents focus on parental depression, even though it is the most common mental illness. Many studies do not address the cumulative losses experienced by children who grow up experiencing depression in a parent. And finally, most studies of children of depressed parents lack a theoretical basis. Pauline Boss's theory (Boss & Greenberg, 1984) has been utilized to explain the experiences and effects on family member's well-being for those patients suffering from a variety of illnesses; however, empirical work with emerging adults who grow up with a mentally ill parent is nonexistent." (p. 216)*

Author, year and country	Study design, participants	Research aims	Recommendations for future research
Mordoch, E. (2010). **Canada**	Interviews with 22 COPMI aged 6 – 12 years (14 boys, 8 girls) who lived full or part time with a parent with a MI. **Youth/children COPMI**	*How do children understand and learn about MI? What do they want to tell other children living with PMI?* (p.20).	*"A volume of research on children of PMI exists but the majority of this research is based on behavioural competence measures and adults' perceptions of children's experiences. Few research studies incorporate children's perspectives. Previous literature focused on risks associated with genetic transmission (Rosenthal, 1970) exposure to parents' pathology (Rutter, 1966) and the effects of multiple risk and protective factors (Werner & Smith, 1992). During the decade of the eighties, the research approach shifted from a disease model to a health promotion model resulting in extensive literature on children's resiliency (Anthony & Cohler, 1987; Garmezy, 1987; Rutter, 1985). However, this literature mainly relied on imposed views of resiliency filtered through adult eyes and focused on behavioural competence which minimized children's subjective experiences (Mordoch & Hall, 2002)." (p.19).*<br>"*Research on when and what to tell children is needed to ensure that all children receive timely and developmentally appropriate information. This study demonstrated children experienced undue hardship when imagining parental health outcomes based on incomplete information*." (p.24)
Morningstar, E. (2013). **USA**	Interviews with 50 young adults between the ages of 19 and 34. **Adult COPMI**	*This dissertation is a qualitative study of the stories young adult children tell about growing up with a parent with a diagnosed mood disorder* (p. iii).	*"Even though research detailing the impact of having a mentally ill parent for children and adults is growing, there is limited research that looks directly at the impact on young adults' lives and more specifically on their transition to adulthood." (p. 1)*<br>*"Life course scholars have emphasized the importance of examining the transitions and trajectories of individuals' lives and the ways in which these lives are linked to others (George 1999; Moen and Hernandez 2009). Despite some work that examines the lives of those who are connected to individuals affected by mental illness, more research in this area is needed to fully understand the impact of mental illness on all who may be affected (George 1999)." (p.2).*

Author, year and country	Study design, participants	Research aims	Recommendations for future research
			"Ultimately, the stories that my respondents told about their parents' illness and the transition to adulthood demonstrate the need for researchers to not only consider the role social attributes such as race, class, gender, and sexuality play in young adults' experiences of the transition to adulthood, but how the types of cultural stories and cultural narratives available to young adults filter the stories they tell." (p.118-119).<br>*"In other words, I was only able to tap into the stories from individuals who identified as a child of a parent with a mood disorder and who saw some benefit and/or felt comfortable talking about their experience. The sibling interviews that I was able to gather suggest that there are children who may think and talk about this experience in very different ways. In fact, there are those who researchers or practitioners would identify as children of parents with mood disorders who may not identify themselves in that way. Future research must consider ways to access this population and whether other research methods may be more helpful in exploring their experiences." (p. 180).*<br>*"Finally, my respondents as a whole could be described as a successful population as the vast majority were currently in college or had already earned at least a bachelor's degree. Their perspectives of the influence of their parent's mood disorder on their lives may be very different from those who faced other challenges or were not embedded in white, middle-class institutions. Future research should examine how children of parents with mood disorders from other class and racial backgrounds understand and explain their experiences and if/how they rely on similar cultural tools." (p. 181)*<br>*"Researchers who study families need to critically examine the role that culture plays in shaping how children understand and talk about their own families. Ideas about what is "normal," "essential," or "harmful" to children and families should not be accepted as a universal truth but as something that shifts over time*

Author, year and country	Study design, participants	Research aims	Recommendations for future research
			and depends on the cultural resources at hand. Rather than treating interviews and surveys of the family as a set of "facts," researchers should use a critical lens to situate their data within the particular cultural context in which it was created. The importance of culture for shaping what matters within people's stories of family should be given more thought and reflection both in terms of research design and data analysis. Researchers should also explore how telling particular stories about families may allow individuals to explain certain ideas of behavior during a particular time in their life course, but may also limit their ability to understand future decisions." (p. 182) "The use of paired-sibling data offers new opportunities for researchers to explore how children within the same family respond to and understand parental mental illness. Sibling data also suggests the importance of gathering stories from multiple perspectives in one family before making claims about the impact of illness, divorce, or other family events." (p. 183).
Nevard, I., Brooks, H., Gellatly, J., Bee, P. (2024) **United Kingdom**	Interviews with 17 COPMI aged 6 – 17 years (6 boys, 11 girls)	*This study aimed to generate inductive findings regarding the phenomenological experiences of COPMI based on an analysis of child generated (rather than proxy generated) data. This study aims to provide a qualitative description of the characteristics, role and function of social networks for COPMI based on an inductive thematic analysis of themes designed to generate network features.*	*"*Network research for COPMI populations is limited [21]*." (p. 2) "There are a number of avenues for future research building upon the theoretical contribution of this study. Further data collection with representative samples of the target demographic of this study will help ascertain the appropriateness of findings across the population as a whole. Further data collection could also improve specificity regarding time frame and pivotal stages in children's developmental career. This is a framework that not only has potential to inform social interventions for COPMI, but one that could with some adjustment be extrapolated into social interventions for young carers as a whole. When contextualised in empirical data that sees increases in adult multi-morbidity across all ages, as well as CYP care load mounting particular in response to the recent pandemic, the coming decade could represent a pivotal time for investing in these interventions [46–49]." (p. 8)*

Author, year and country	Study design, participants	Research aims	Recommendations for future research
Nolte, L., & Wren, B. (2016). **United Kingdom**	Interviews with 15 parents (13 mothers and 2 fathers) all had been diagnosed with severe and enduring mental health difficulties. **Parents**	*The aim of this study was to explore how parents think about and experience talking to their children about their MHP*. (p. 6).	*"Future research. Despite these limitations this study provides a richer understanding of the social processes that impact on parents' decisions about whether to talk to their children about PMHP or remain silent. Given the clear evidence for the importance of understanding and meaning-making as protective factor for children where there are PMHP, further research that helps us understand family dynamics around talking and remaining silent is indicated". (p.29).*
O'Brien, L., Anand, M., Brady, P., & Gillies, D. (2011). **Australia**	Interviews with 9 clinicians (3 nurses, 2 psychiatrists/ registrars, 2 social workers, and 2 occupational therapists) who had worked at acute units for at least the previous 12 months. **Clinicians.**	*The aim of this project was to gain a better understanding of the experiences, barriers, and needs of children, their parents and carers, and staff in order to understand the experience of children visiting their parent in an acute care mental health facility…The focus of this paper is the perceptions of staff regarding children visiting an acute mental health facility*. (p. 2).	*"There is worldwide recognition that separation between parents and children should be minimized during the parent's hospitalization for a mental illness, and that there is a need for 'family-friendly' visitor facilities (AICAFMHA 2004; Howard 2000; O'Shea et al. 2004; Scott et al. 2007), but at this time, there appears to be little done to facilitate children visiting (Howard 2000; O'Shea et al. 2004; Ramchandani & Stein 2003),and there is no research to inform how it should be supported." (p.2).*
Power, J., Goodyear, M., Maybery, D., Reupert, A., O'Hanlon, B., Cuff, R., & Perlesz, A. (2016). **Australia**	Interviews with 11 participants' (8 women) ages ranged from 18 to 51 years. **Adult COPMI**	*In this article we explore how family resilience can be conceptualised in families where a parent has a mental illness. Through a series of qualitative interviews with adults who were raised in a household with a parent who had a mental illness, we aim to explore the experiences of these families with a view to understanding the ways in which individuals and families coped with the challenges associated with parental mental illness.* (p. 70).	*"There is little focus on the impact of shame or stigma in existing family resilience literature. This study emphasises the relevance of this for future research in both family resilience and mental illness." (p. 79).*

Author, year and country	Study design, participants	Research aims	Recommendations for future research
Radley, J., BarNo, J., & Johns, L. (2023).	Interviews with 5 parents with a psychotic disorder, 4 children, 3 partners and one grandmother. **Parents, parners, family members, COPMI**	The aim of this study was to understand how an episode of psychosis affects a parent and their family by procreation or choice, and in what ways the experiences of family members are similar or dissimilar. (p.349)	*"It is important that research continues to try to identify the benefits of such [to support parents in talking about their mental health to their children] interventions and that any explanations about parental mental illness are age-appropriate. More work needs to be done to investigate how to reassure and motivate parents with psychotic experiences and other adults in the family to talk to the children, and provide these families with developmentally appropriate explanations of psychosis". (p.359)*
Reupert, A. E., & Maybery, D. (2010). **Australia**	Interviews with 18 Expert COPMI program facilitators (Clinicians) from a Australian national public database of COPMI programs. **COPMI program facilitators**	*Thus, in this study we sought to identify, from those working in this area, why they might educate COPMI about their parent's mental illness (or why not), how they educate children (for instance, video, lecture style or discussion groups), what they believed COPMI needed to know about their parent's mental illness and how education might differ, according to different subgroups of COPMI (such as different age groups).* (p. 633).	*"Additionally, clinical trials are required that isolate the education component of COPMI programs and determine their effectiveness in intervening with this target group in terms of enhancing knowledge. Such studies might also consider the impact of enhanced knowledge on a variety of well-being measures. (p.641). They suggest different ways of discussing parental illness and hospitalization (for physical not mental illness) for children aged 6 to 11 years of age, and then for those aged 12 and over, that might be further explored in the context of parental mental illness.(p. 642). Future studies need to compare different educational modes, for example, family psycho-education and peer group discussions and ascertain which is the most effective mode of education, in terms participant outcome and cost (including both time and money). The view that "knowledge is power" provides relief and offers young people the language to discuss their circumstances with others has been highlighted and provides one model of education, which needs to be investigated in future studies and clinical settings". (p.643).*
Tabak, I., Zabłocka-Zytka, ˙ L., Ryan, P., Poma, S. Z.,	Focus groups and interviews conducted with n=96 **clinicians** (n=50), **parents with mental illness**	*The research questions for this study were: 1 What are the perceptions of professionals and stake holders experiencing mental illness in their*	No recommendations for future research

Author, year and country	Study design, participants	Research aims	Recommendations for future research
Joronen, K., Vigano, ` G., … Dawson, I. (2016). **International (see design)**	(n=31), **adult children and partners** (n=31) of parents with mental illness from England, Finland, Germany, Italy, Norway, Poland and Scotland. **Parents Adult COPMI Partners Clinicians**	*family regarding the main consequences for children growing up in these families, the main needs of these children, and the main expectations of these children from professionals and school?* *2 Are there any differences in these perceptions between different stakeholder groups and countries?* (p. 321).	
Tanonaka, K., & Endo, Y. (2021). **Japan**	Interviews with 10 Adults (20-40 years) who were children of parents with mental illness. **Adult COPMI**	*This study thus aimed to elucidate the resources recognized as helpful by COPMI to cope with the difficulties they experienced in their lives….Resources may be external (i.e., supports) or internal to the individual child (i.e., inner resources). Furthermore, we sought to identify the wide range of resources recognized by children that may have helped with their emotional and daily needs, which may include parental and teacher supports, adult mentoring, community organizations, and children's strengths that promote positive child development*. (p.2).	*"However, despite the variety of challenges faced by these children, scant research has investigated the details of interventions targeting the daily life difficulties of COPMI. Further, few studies have examined these challenges from the child's perspective (Gladstone, Boydell, & McKeever, 2006; Mordoch, 2010). Analyses performed from the perspectives of experts alone do not adequately assess whether the interventions are serving children's real needs." (p.2).* *"Future studies should survey minors with parents of various mental illnesses to investigate the types of care and support needed according to the situation or parent's disorder." (p.10)*
Trondsen, M. V. (2012). **Norway**	600 posts - online COPMI self-help group, from 16 (15 girls), 15-18 year olds. **Youth COPMI**	*…in this article I focus on the perspectives of children and adolescents and their experiences of living with a mentally ill parent by exploring the*	*"Bearing these considerations in mind, my main argument in this article is that to increase the knowledge about parental mental illness it will be useful to supplement the existing risk and resilience research with studies that explore children's and adolescents' own perspectives and experiences in their everyday*

Author, year and country	Study design, participants	Research aims	Recommendations for future research
		communication within an online self-help group for adolescents. (p.176).	*lives. Taking their voices into account provides valuable insights into perspectives that differ from those of parents or health professionals. I also suggest the importance of immediate attention to the context of family situations and the complexity of children's and adolescents' everyday lives with a mentally ill parent." "The participants were also self-recruited, with no restriction on gender, and only one boy chose to participate. I suggest that the gender issue should be explored in future research about participation in online and offline self-help groups, and when addressing the everyday experiences of children and adolescents with mentally ill parents." "In accordance with previous research (Fjone et al.,2009; Gladstone et al., 2006; Mordoch & Hall, 2008), I argue for the importance of including the perspectives of children and adolescents in research on parental mental illness. Taking their perspectives into consideration will make their experiences more visible and provide important means to improve health care for families living with mental illness." (p. 185).*
Trondsen, M., & Tjora, A. (2014). **Norway**	Interviews with n=13 participants of an online self-help group for adolescent girls (15-18) with a parent with mental illness. **Youth COPMI**	*We explored the role of a Norwegian online self-help group for adolescents (aged 15 to 18) with a mentally Ill parent.* (p. 1407). *We explore how Internet use is embedded in a wider everyday context, as one of many sources of information and support, and with significant potential relevance for life as such. Accordingly, the research design included everyday experiences of adolescents with a mentally ill parent, moving into the details of how participation in an online forum was related to "offline" experiences*. (p. 1408).	*"the body of research on online self-help groups has focused primarily on services for adult users and for adult caregivers of a child/partner suffering from illness." "...more research is needed to determine the usefulness of online information and support for caregivers (Gage & Panagakis). Moreover, children's and adolescents' perspectives are often different from those of adults (Mordoch, 2010). Studies of online support interventions for children and adolescents living with parental mental illness are nearly nonexistent (Drost et al., 2011)." (p.1408)*

Author, year and country	Study design, participants	Research aims	Recommendations for future research
Van Parys, H., & Rober, P. (2013). **Belgium**	Interviews with 15 families with children between 7 and 14 years old participated in family (ill parent, children and sometimes partners) also in interviews. **Youth COPMI AND FAMILY**	*How do children experience parental depression and how do they experience their own caregiving in the family?* (p. 332).	*"The relative lack of rich verbalized experiential data might be related to the context of the interview: children might have been more reluctant to talk about their experience in the presence of their parent. Individual interviews or focus groups with children would give access to a different set of data, and as a result would give us an additional perspective on childhood experiences." (p.341).* *"While analyzing family interviews, we learned to be careful not to overlook silences and other nonverbal cues. But many things are left unspoken and therefore the question remains: How to research concepts like "hiding worry" in an empirical way? Studying what is hidden or implicit is difficult because as a researcher you understand the words in a certain context, including complex nonverbal cues. Whereas words are compelling in the context of research, the nonverbal issues are sometimes difficult to express in an article, let alone to objectify them." (p.342) "…our interpretation should be handled with caution and needs further exploration, for instance in other qualitative research projects." (p. 342)*
Van Parys, H., Bonnewyn, A., Hooghe, A., De Mol, J., & Rober, P. (2015). **Belgium**	Focus groups with 18 women and 3 men, 21 to 29 year-olds who grew up with a depressed parent. **Adult COPMI**	First, we set out to determine how young adults make sense of their childhood experiences of parental depression. Second, we explored how their retrospective reflections could enable us to understand the experiences of children in processes of parentification. (p. 524).	"*In a social constructionist view, participants in a focus group construct their narrative together (Daly, 2007). In two focus groups, the participants explicitly referred to the beneficial experience of this retrospective meaning making together with others who grew up with a parent with depression. Some authors have argued that the actual interaction in focus groups should be taken into account when analyzing the data (Halkier, 2010; Markova, Linell, Grossen, & Orvig, 2007; Onwuegbuzie, Dickinson, Leech, & Zoran, 2009; Wilkinson, 2003). Microanalysis of one or more of these focus groups can be considered as a suggestion for future research.*" (p. 534).
Vetri, K., Piché, G.,	Qualitative survey among 8 parents, 8 children, and 6	The present study aims to evaluate the IPT-based book Le Trésor de l'Île Rouge targeting elementary school-aged	*"More broadly, the involvement of children themselves in the evaluation of interventions ensures the evaluations are accessible, relevant and appreciated, which then ensures that*

Author, year and country	Study design, participants	Research aims	Recommendations for future research
Villatte, A. (2022).	psychosocial workers. **COPMI, parents, clinicians**	children with a parent with a mental illness.<br>The formative evaluation examined children, parents and psychosocial workers perception of the acceptability, appropriateness and utility of the book. (p.3)	*the services made available to them are actually used (50). P.9 Children's views of the content and modalities proposed in intervention programs developed to support them are rarely collected, despite the importance (72) and numerous benefits (73) of doing so. Their views may often differ from those of parents or psychosocial workers (44). Data from research using this type of design are known to be more valid and accurate because they come from the children themselves, the primary target audience (73, 74). Children are in the best position to share their perspectives (ideas, opinions, suggestions) and experiences (75, 76)". P.9*<br>*"This research highlights the importance of adapting the content and modalities of interventions to children's interests, preferences, needs and developmental level. Future research should focus on promoting children's participation in the research process. It's important to develop a research culture where children's voices are heard and their involvement in the activities and decisions affecting them is an integral part of everyday research practice". Pp.9-10*
Villatte, A., Piché, G., & Benjamin, S. (2021).	Photovoice group meetings with 10 young adults (18-15 years old) that have at least one parent with a mental illness. **COPMI**	The present study pursues two main objectives: (1) identify which aspects of social support youth whose parents have a mental illness during their transition to adulthood spontaneously address when talking about their experiences in Photovoice workshops; (2) explore how participants view these types of workshops as a good way to improve their sense of social support and belonging.	*"In addition to being few in number, studies that have considered the perspectives of youth of parents with a mental illness transitioning to adulthood on the social support they provide and receive all adopt "traditional" research designs by interviewing youth using questionnaires or interviews, with the limitation of restricting their responses by pre-established questions. The value of using participatory research methods that focus on artistic mediums-such as the Photovoice method that relies on photography and storytelling (56)-has been underlined with other clienteles, particularly in the mental health field (57, 58). Participating in this type of research stimulates participants' reflections and expression while allowing them to become more aware of the recurring issues they encounter, to consider solutions that make sense collectively, and to feel that they are contributing*

Author, year and country	Study design, participants	Research aims	Recommendations for future research
			to social change by producing data that will be brought to the attention of decision-makers". (P.3)<br><br>*"More research is needed to clarify the nature and strength of the links between the perception of positive nonparental social support and the development of resilience in young adults with a parent with a mental illness". (p.11)*<br>*"On another note, some dimensions of social support were not examined in this study (59). Although the SPS-10 has excellent psychometric qualities (63), it would be beneficial to combine its use with instruments assessing the diversity and size of the network, as well as the quality of relationships (84) in order to capture the multiple facets of the concept of social support in future studies". (p.12)*
Widemalm, M., & Hjarthag, ¨ F. (2015). **Sweden**	301 comments from 35 forum threads on 5 different Swedish Internet forum for COPMI – Mean= 22 years, between 13 to 49 years. **Youth COPMI**	*The aim of this study was to identify how daughters or sons to parents suffering from mental illness perceive their situation. The objective was to provide new knowledge based on what they communicate on open Internet forums*. (p. 1601).	*"The role of social support and the attractiveness of anonymity and availability typical for open Internet forums ought to be considered by health care professionals and researchers when developing new ways for providing support for children or adolescents with a mentally ill parent." (p.1601) / "To conclude, we urge health care professionals and researchers to consider both the role of social support from peers and the attractiveness of anonymity and availability of open Internet forums when developing new support interventions for children or adolescents with a mentally ill parent." (p.1606)*<br>*"There are some limitations when studying open Internet forum threads this way. It cannot be controlled for, or guaranteed, that the forum writers really had parents with a psychiatric diagnosis since they were anonymous. Hence, we cannot be sure about the writers' age or sex, how honest they were when writing, or what their motives for writing really were. Research is still scarce, which is why our results should be followed up in future studies using method triangulation to learn more about the role that open*

Author, year and country	Study design, participants	Research aims	Recommendations for future research
			Internet forums has for people with a mentally ill parent." (p. 1606).

References

1. Bosch, A., Riebschleger, J., & van Loon, L. (2017). Dutch youth of parents with a mental illness reflect upon their feelings of guilt and shame. International Journal of Mental Health Promotion, 19(3), 159-172.
2. Fjone, H. H., Ytterhus, B., & Almvik, A. (2009). How children with parents suffering from mental health distress search for 'normality' and avoid stigma: To be or not to be... is not the question. Childhood, 16(4), 461–477
3. Foster, K., Lewis, P., & Mccloughen, A. (2014). Experiences of peer support for children and adolescents whose parents and siblings have mental illness. Journal of Child and Adolescent Psychiatric Nursing, 27(2), 61–67. https://doi.org/10.1111/jcap.12072
4. Fudge, E., & Mason, P. (2004). Consulting with young people about service guidelines relating to parental mental illness. Australian e-journal for the Advancement of Mental Health, 3(2), 50-58.
5. Knutsson-Medin L, Edlund B & Ramklint M (2007) Experiences in a group of grown-up children of mentally ill parents. Journal of Psychiatric and Mental Health Nursing 14, 744–752.
6. Trondsen, M. V. (2012). Living with a mentally ill parent: Exploring adolescents' experiences and perspectives. Qualitative Health Research, 22(2),174–188. https://doi.org/10.1177/1049732311420736
7. Trondsen, M., & Tjora, A. (2014). Communal normalization in an online self-help group for adolescents with a mentally ill parent. Qualitative Health Research, 24(10), 1407–1417. https://doi.org/10.1177/1049732314547708
8. Van Parys, H., & Rober, P. (2013). Trying to comfort the parent: A qualitative study of children dealing with parental depression. Journal of Marital and Family Therapy, 39(3), 330–345.
9. Widemalm, M., & Hjarthag, ̈ F. (2015). The forum as a friend: Parental mental illness and communication on open Internet forums. Social Psychiatry and Psychiatric Epidemiology, 50(10), 1601–1607.
10. Mordoch, E. (2010). How children understand parental mental illness: "You don't get life insurance. What's life insurance?". Journal of the Canadian Academy of Child and Adolescent Psychiatry, 19(1), 19.
11. Tanonaka, K., & Endo, Y. (2021). Helpful resources recognized by adult children of parents with a mental illness in Japan. Japan Journal of Nursing Science, e12416.
12. Van Parys, H., Bonnewyn, A., Hooghe, A., De Mol, J., & Rober, P. (2015). Toward understanding the child's experience in the process of parentification: Young adults' reflections on growing up with a depressed parent. Journal of Marital and Family Therapy, 41(4), 522–536.
13. Power, J., Goodyear, M., Maybery, D., Reupert, A., O'Hanlon, B., Cuff, R., & Perlesz, A. (2016). Family resilience in families where a parent has a mental illness. Journal of Social Work, 16(1), 66-82.
14. Morningstar, E. (2013). Transitions in Turmoil? Young adult children of parents with mood disorders and the transition to adulthood (Doctoral dissertation). Retrieved from ProQuest Dissertations and Theses. (3562022).
15. Mechling, B. (2016). From shadows to hope: Shared experiences of emerging adults who grew up with a depressed parent in the home. Issues in Mental Health Nursing, 37(4), 211–218.
16. Duncan G & Browning J (2009) Adult attachment in children raised by parents with schizophrenia. Journal of Adult Development 16, 76–86

17. Drost, L. M., & Schippers, G. M. (2015). Online support for children of parents suffering from mental illness: A case study. Clinical Child Psychology and Psychiatry, 20(1), 53-67.
18. Bartsch, D. R., Roberts, R. M., Davies, M., & Proeve, M. (2016). Understanding the experience of parents with a diagnosis of borderline personality disorder. Australian Psychologist, 51(6), 472-480.
19. Marston, N., Maybery, D. & Reupert, A. (2014). Empowering families where a parent has a mental illness: A preliminary evaluation of the 'family focus' DVD, Advances in Mental Health (2014) 12(2): 136–146.
20. Nolte, L., & Wren, B. (2016). Talking or keeping silent about parental mental health problems—A grounded theory of parents' decision making and experiences with their children. Journal of Marital and Family Therapy, 42(4), 731–744.
21. Afzelius, M., Plantin, L., & Ostman, M. (2018). Families living with parental mental illness and their experiences of family interventions. Journal of Psychiatric and Mental Health Nursing, 25(2), 69-77.
22. Maybery, D., Reupert, A., & Goodyear, M. (2015). Goal setting in recovery: families where a parent has a mental illness or a dual diagnosis. Child & Family Social Work, 20(3), 354-363.
23. Isobel, S., Pretty, D., & Meehan, F. (2017). 'They are the children of our clients, they are our responsibility': a phenomenological evaluation of a school holiday program for children of adult clients of a mental health service. Advances in Mental Health, 15(2), 132-146.
24. Hoadley, B., Falkov, A., & Agalawatta, N. (2019). The acceptability of a single session family focused approach for children/young people and their parents attending a child and youth mental health service. Advances in Mental Health, 17(1), 44-54.
25. Tabak, I., Zabłocka-Zytka, ˙ L., Ryan, P., Poma, S. Z., Joronen, K., Vigano, ` G., … Dawson, I. (2016). Needs, expectations and consequences for children growing up in a family where the parent has a mental illness. International Journal of Mental Health Nursing, 25(4), 319–329. https://doi.org/10.1111/inm.12194.
26. Bartsch, D. R., Roberts, R. M., Davies, M., & Proeve, M. (2015). Borderline personality disorder and parenting: clinician perspectives. Advances in Mental Health, 13(2), 113-126.
27. O'Brien, L., Anand, M., Brady, P., & Gillies, D. (2011). Children visiting parents in inpatient psychiatric facilities: Perspectives of parents, carers, and children. International Journal of Mental Health Nursing, 20(2), 137–143.
28. Reupert, A. E., & Maybery, D. (2010). "Knowledge is power": Educating children about their parent's mental illness. Social Work in Health Care, 49(7), 630-646.
29. Grove, C., Riebschleger, J., Bosch, A., Cavanaugh, D., & van der Ende, P. C. (2017). Expert views of children's knowledge needs regarding parental mental illness. Children and Youth Services Review, 79, 249-255.
30. Tussing, H., & Valentine, D. (2001). Helping adolescents cope with the mental illness of a parent through bibliotherapy. Child & Adolescent Social Work Journal, 18(6), 455–469.
