# Supplementary material for: Exploring past research to move forward: a scoping review of aims, outcomes, and recommendations in parental mental illness qualitative research
Source: Front Public Health. 2024 Oct 16;12:1427432. doi: 10.3389/fpubh.2024.1427432 (PMC11521813; doi:10.3389/fpubh.2024.1427432)
Supplement: Supplementary file 2 [file Table_2.pdf]

Supplementary Table 2. Quality assessment of the qualitative studies with the CASP Critical Appraisal Skills Checklist

Items / Articles	Total scores	1	2	3	4	5	6	7	8	9	10
Afzelius, M., Plantin, L., & Ostman, M. (2018). **Sweden**	17 Moderate	High	High	No	High	High	Somewhat	High	High	High	High
Bartsch, D. R., Roberts, R. M., Davies, M., & Proeve, M. (2016). **Australia**	17 Moderate	High	High	High	Somewhat	High	Somewhat	High	High	Somewhat	High
Bartsch, D. R., Roberts, R. M., Davies, M., & Proeve, M. (2015). **Multi-country**	13 Low	High	High	High	Somewhat	High	No	No	Somewhat	Somewhat	High

Bosch, A., Riebschleger, J., & van Loon, L. (2017). **The Netherlands**	17 Moderate	High	High	High	High	High	No	Somewhat	High	High	High
Cudjoe, E., Tam, C., Effah, D., Amegashie, E., & Tweneboah, A. (2022). **Ghana**	18 Moderate	High	High	High	High	High	Somewhat	High	High	Somewhat	High
Cudjoe, E., Tam, C., & Chiu, M. (2023). **Ghana**	16 Moderate	High	High	High	High	High	No	High	Somewhat	Somewhat	High
Dean, L., Buechner, H., Moffett, B., Maritze, M., Dalton, L., Hanna, J., Rapa, E., Stein, A.,	17 Moderate	High	High	High	High	Somewhat	Somewhat	High	Somewhat	High	High

Tollman, S., & Kahn, K. (2023). **South Africa**											
Drost, L. & Schippers, G. (2015). **The Netherlandas**	18 Moderate	High	High	High	Somewhat	High	Somewhat	High	High	High	High
Duncan, G. & Browning, J. (2009). **New Zealand**	14 Low	High	High	High	Somewhat	High	No	Somewhat	No	High	High
Fjone, H., Ytterhus, B., & Almvik, A. (2009). **Norway**	6 Low	High	High	No	No	High	No	No	No	No	No
Foster, K., Lewis, P., & Mccloughen, A. (2014). **Australia**	16 Moderate	High	High	High	Somewhat	Somewhat	No	High	High	High	High

Grove, C., Riebschleger, J., Bosch, A., Cavanaugh, D., & van der Ende, P. C. (2017). **Multi-country**	16 Moderate	High	High	High	High	High	No	High	High	Somewhat	Somewhat
Harries, C. Smith, D., Gregg, L., Allott, R., & Wittkowski, A. (2023). **United Kingdom**	18 Moderate	High	High	High	High	Somewhat	Somewhat	High	High	High	High
Hoadley, B., Falkov, A., & Agalawatta, N. (2019). **Australia**	11 Low	High	High	High	No	No	No	Somewhat	No	High	High

Isobel, S., Pretty, D., & Meehan, F. (2017). **Australia**	18 Moderate	High	High	High	High	High	No	High	High	High	High
Knutsson-Medin, L., Edlund, B., & Ramklint, M. (2007). **Sweden**	12 Low	High	High	No	High	Somewhat	No	No	High	High	Somewhat
Marston, N., Maybery, D. & Reupert, A. (2014). **Australia**	12 Low	High	High	No	High	Somewhat	Somewhat	No	No	High	High
Maybery, D., Reupert, A., & Goodyear, M. (2015). **Australia**	12 Low	High	High	No	Somewhat	High	Somewhat	No	High	Somewhat	Somewhat
Mechling, B. (2016). **USA**	13 Low	High	High	High	High	Somewhat	No	High	No	Somewhat	Somewhat

Mordoch, E. (2010). **Canada**	18 Moderate	High	High	High	High	High	Somewhat	High	High	High	Somewhat
Morningstar, E. (2013). **USA**	15 Low	High	High	No	High	High	Somewhat	Somewhat*	High	Somewhat	High
Nevard, I., Brooks, H., Gellatly, J., Bee, P. (2024) **United Kingdom**	18 Moderate	High	High	High	High	High	Somewhat	Somewhat	High	High	High
Nolte, L., & Wren, B. (2016). **United Kingdom**	15 Low	High	High	No	High	High	No	Somewhat	High	High	High
O'Brien, L., Brady, P., Anand, M., & Gillies, D. (2011). **Australia**	13 Low	High	High	High	Somewhat	High	No	High	No	Somewhat	Somewhat

Power, J., Goodyear, M., Maybery, D., Reupert, A., O'Hanlon, B., Cuff, R., & Perlesz, A. (2016). **Australia**	12 Low	High	High	No	Somewhat	High	No	Somewhat	High	Somewhat	Somewhat
Radley, J., BarNo, J., & Johns, L. (2023). **United Kingdom**	16 Moderate	High	High	High	High	Somewhat	No	High	High	Somewhat	High
Reupert, A. E., & Maybery, D. (2010). **Australia**	13 Low	High	High	No	High	Somewhat	No	Somewhat	Somewhat	High	High
Tabak, I., Zabłocka-Zytka, ˙ L., Ryan, P., Poma, S. Z., Joronen, K., Vigano, ` G., …	14 Low	High	High	No	Somewhat	High	No	High	High	High	Somewhat

Dawson, I. (2016). **Multi-Country**											
Tanonaka, K. & Endo, Y. (2021). **Japan**	17 Moderate	High	High	High	Somewhat	Somewhat	Somewhat	High	High	High	High
Trondsen, M. V. (2012). **Norway**	20 High	High	High	High	High	High	High	High	High	High	High
Trondsen, M., & Tjora, A. (2014). **Norway**	18 Moderate	High	High	High	High	High	Somewhat	High	High	Somewhat	High
Van Parys, H., Bonnewyn, A., Hooghe, A., De Mol, J., & Rober, P. (2015). **Belgium**	18 Moderate	High	High	High	High	High	No	High	High	High	High
Van Parys, V. & Rober, P. (2013). **Belgium**	19 Moderate	High	High	High	High	High	Somewhat	High	High	High	High

Vetri, K., Piché, G., Vilatte, A. (2022). **Canada**	18 Moderate	High	High	High	High	High	Somewhat	High	Somewhat	High	High
Villatte, A., Piché, G., & Benjamin, S. (2021). **Canada**	18 Moderate	High	High	High	High	High	No	High	High	High	High
Widemalm, M. & Hjärthag, F. (2015). **Sweden**	12 Low	High	High	No	High	High	No	No	High	Somewhat	Somewhat

Note. *Critical Appraisal Skills Programme (2018). CASP Qualitative Studies Checklist. [online] Available at: https://casp-uk.net/images/checklist/documents/CASP-Qualitative-Studies-Checklist/CASP-Qualitative-Checklist-2018.pdf. Accessed: June 3 2023.*
